# Dissection of the genetic basis of genotype-by-environment interactions for grain yield and main agronomic traits in Iranian bread wheat landraces and cultivars

**DOI:** 10.1038/s41598-021-96576-1

**Published:** 2021-09-07

**Authors:** Hadi Alipour, Hossein Abdi, Yousef Rahimi, Mohammad Reza Bihamta

**Affiliations:** 1 Department of Plant Production and Genetics, Faculty of Agriculture, Urmia University, Urmia, Iran; 2 Department of Plant Biology, Swedish University of Agricultural Sciences, Uppsala, Sweden; 3 Department of Agronomy and Plant Breeding, College of Agriculture and Natural Resources, University of Tehran, Karaj, Iran

**Keywords:** Biotechnology, Genetics, Plant sciences

## Abstract

Understanding the genetic basis of performance stability is essential to maintain productivity, especially under severe conditions. In the present study, 268 Iranian bread wheat landraces and cultivars were evaluated in four well-watered and two rain-fed conditions for different traits. According to breeding programs, cultivars were in a group with a high mean and stability in terms of GY, GN, and SW traits, while in terms of PH, they had a low mean and high stability. The stability of cultivars and landraces was related to dynamic and static stability, respectively. The highest number of marker pairs and lowest LD decay distance in both cultivars and landraces was observed on the B genome. Population structure differentiated indigenous cultivars and landraces, and the GWAS results for each were almost different despite the commonalities. Chromosomes 1B, 3B, 7B, 2A, and 4A had markers with pleiotropic effects on the stability of different traits. Due to two rain-fed environments, the Gene Ontology (GO) confirmed the accuracy of the results. The identified markers in this study can be helpful in breeding high-performance and stable genotypes and future breeding programs such as fine mapping and cloning.

## Introduction

Bread wheat (*Triticum aestivum* L.) is the most important cereal for humans and has an undeniable role in food security. Therefore, increasing grain yield and yield stability have been prioritized by breeding programs to maintain wheat productivity. However, such a goal is challenged by the genotype-by-environment interaction (GEI) because a polygenic attribute like grain yield is controlled by numerous major and minor effect genes that interact with each other and the environment^1,2^. Thus, genotypes usually show a wide range of reactions before being introduced in a multi-environment trail (MET), leading to changes in their performance rankings and thus more confusion for breeders.

Yield stability may be obtained through a combination of agronomic traits^3^. Even though wheat grain yield stability has been specifically studied so far, in recent years, some studies have correctly focused on the GEI pattern in yield components^4–7^. In cereals, grain yield can be affected by yield components directly or indirectly. However, indirect improvement of yield stability might not be possible through agronomic traits^8^. This is due to the complex nature of performance stability controlled by genetic factors^9^ and can be interpreted using genotypic and environmental covariables^10,11^.

Stability statistics with almost simple calculation operations have long been the most important methods for assessing the stability of genotypes. Such statistics usually have a clear interpretation and can cover various aspects of stability, including static and dynamic types. Stability in the static concept refers to the constant performance of the genotype in different environments. In contrast, stability in the dynamic concept is the performance of the genotype that is constant according to the estimated or predicted level of the environments. These concepts are equivalent to biological and agronomic stability, respectively^12,13^. However, obtaining a genotype that maintains its yield in all environments is almost impossible, and such a concept of stability is not appropriate for production as genotypes are expected to behave well under favorable environmental conditions. On the other hand, the performance of stable genotype with a dynamic concept in response to different environments is parallel to the average response of all studied genotypes^8^. Therefore, the use of dynamic stability during breeding programs leads to increased resilience to climate change in new varieties^14^. So far, about 50 different stability statistics, both parametric and non-parametric, have been used. Woyann et al.^15^ stated that Wricke’s^16^ ecovalance (Wi) and additive main effects and multiplicative interaction (AMMI) statistics, namely AMMI stability value (ASV)^17^ and Modified AMMI stability index (MASI)^18^, emphasize stability, while Finlay and Wilkinson^19^ regression coefficient (bi) measures adaptability. In other words, Wi measures dynamic stability^20^, while low values of bi are estimates of static stability^8^. The method of harmonic mean of the relative performance of the genetic values (HMRPGV) provides estimates of adaptability and genotypic stability based on mixed models^21^. Several statistics have simultaneously examined performance and stability, including the yield stability index (YSI)^22^ and weighted average of absolute scores from the singular value decomposition of the matrix of BLUP for the GEI effects generated by an LMM and response variable (WAASBY) index^23^. In one of the latest statistics, while emphasizing different traits in MET analysis, the multi-trait stability index was introduced^24^.

Different chromosomal regions are involved in wheat adaptation^25^. Determining molecular markers associated with quantitative traits and indices of trait stability and adaptability can help identify regions of the genome that control GEI^26^. Furthermore, identifying genomic regions that affect stability can facilitate the selection process^27^. In addition, understanding the interaction of QTL-by-environment is also important because most related QTLs are not stable across environments, and the repeatability of marker-trait associations (MTA) is widely disturbed by the GEI^28^. MTAs have been identified for the stability index on chromosomes 4B and 7B^29^. In a genetic architecture study, the grain yield stability of wheat and other traits using the GWAS approach identified several SNPs on different chromosomes that affected their mean traits and stability^9^. In addition, the role of functional markers, including photoperiod genes, in performance stability has been revealed^14^. The combination of GWAS and genomic prediction suggested that dissecting the genetic basis of yield stability would be more complex than the one in grain yield^29^. Other similar studies identified stability-related QTLs in the barley^20,26,30^, soybean^27^, and rice^31^. However, there are a few studies on the dissection of GEI using genome-wide association studies in wheat. The present study investigated the stability of Iranian bread wheat in terms of different traits and diversity indices of SNP markers. Then, to understand the genetic basis of GEI, we used association analysis for stability indices and examined the ontology of the identified genes.

## Results

### Genotype-by-environment interaction

**Figure 1:**
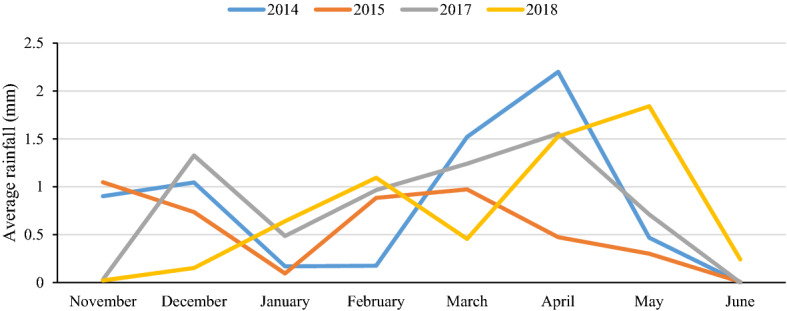
Average rainfall in different months each year.

**Figure 2:**
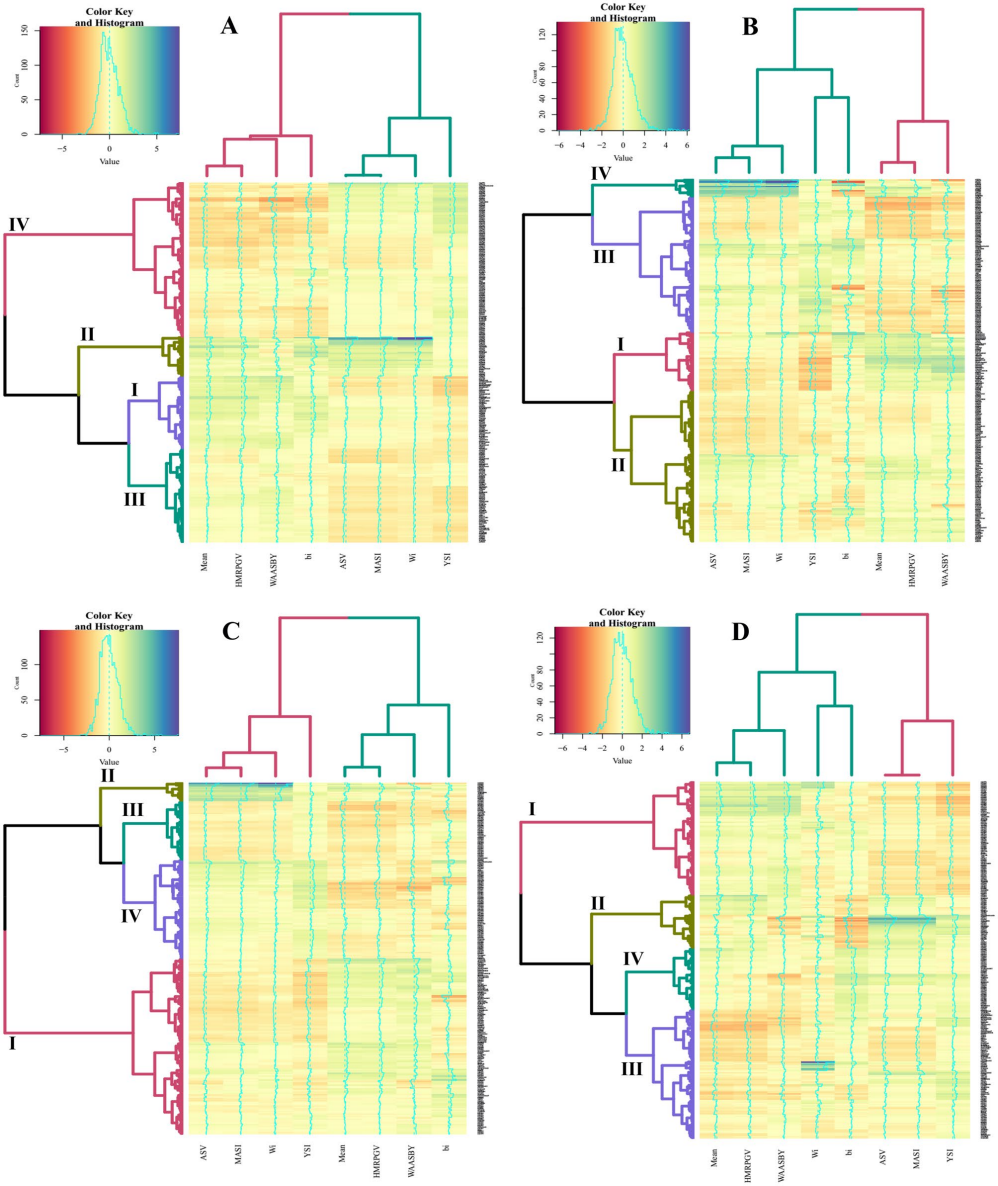
Heatmap based on stability indicators for grain yield (**A**), number of grains (**B**), spike weight (**C**), and plant height (**D**) in Iranian wheat landraces and cultivars. I: high mean and stable, II: high mean and unstable, III: low mean and stable and IV: low mean and unstable. Mean: average trait in all environments, ASV: AMMI stability value, b_i_: Finlay-Wilkinson regression, W_i_: Wricke’s ecovalance measures, MASI: Modified AMMI stability index, YSI: Yield stability index, HMRPGV: harmonic mean of the relative performance of the genetic values, WAASBY: weighted average of absolute scores from the singular value decomposition of the matrix of BLUP for the GEI effects generated by an LMM and response variable. The heatmaps were created using "gplots" package "heatmap.2" function in R^32^.

The effects of genotype, environment, and GEI were significant at different probability levels for the four traits in the total population and subpopulations (Table 1). Due to drought stress in the study and different rainfall patterns in different years (Fig. 1), such a result was not unexpected. Broad sense heritability was low for GY, moderate for SW and GN, but high for PH. To select the desired genotypes in terms of mean traits and stability, we used different statistics, and the results are presented in Fig. 2. Based on these two criteria, genotypes were divided into approximately four classes: (I) high mean and stable, (II) high mean and unstable, (III) low mean and stable, and (IV) low mean and unstable. In terms of GY, 54, 29, 70, and 115 genotypes were present in these classes, respectively. Cultivars included 37.5%, 9.1%, 36.4%, and 17%, and landraces included 11.7%, 11.7%, 21.1% and 55.5% of the members of these classes, respectively (Fig. 2A). In terms of GN, in class I 43 genotypes (38.6% of cultivars and 5% of landraces), in class II 113 genotypes (42% of cultivars and 42.2% of landraces), in class III 99 genotype (14.8% of cultivars and 47.8% of landraces), and in class IV 13 genotypes (4.5% of cultivars and 5% of landraces) were present (Fig. 2B). According to SW, 135 genotypes (79.5% of cultivars and 36.1% of landraces), 13 genotypes (3.4% of cultivars and 5.5% of landraces), 45 genotypes (6.8% of cultivars and 21.7% of landraces), and 66 genotypes (10.2% of cultivars and 36.7% of landraces), were observed in the mentioned four classes, respectively (Fig. 2C). For plant height, there were 85 genotypes (19.3% of cultivars and 37.8% of landraces) in class I, 40 genotypes (10.2% of cultivars and 17.2% of landraces) in class II, 97 genotypes (62.5% of cultivars and 23.3% of landraces) in class III, and 46 genotypes (8% of cultivars and 21.7% of landraces) in class IV (Fig. 2D). On the other hand, as expected, some indices, especially HMRPGV and WAASBY, were correlated with the mean of the traits and were in the same group (Fig. 2). Since it is difficult to select genotypes with simultaneous stability for all four traits, we calculated the multi-trait stability index based on yield and yield components (Supplementary Fig. 1). The results interestingly showed that 11 cultivars (12.5%) and 29 landraces (16.1%) formed the genotype selected based on this index (Supplementary Table 3).

**Table 1:** Mean, standard deviation (SD), broad sense heritability (H^2^), and combined analysis of variance based on studied traits in 286 Iranian wheat landraces and cultivars and 6 environments.

Abb	Trait	Group	Mean	SD	H^2^	Mean squares			
						Env	Rep (Env)	Gen	Env × Gen
GY	Grain yield (g/plant)	Total	1.802	0.892	0.418	***	**	***	***
		Landrace	1.713	0.869	0.354	***	*	***	***
		Cultivar	1.982	0.912	0.312	***	**	***	***
GN	Grain number	Total	38.72	10.82	0.696	*	***	***	***
		Landrace	36.64	10.19	0.594	**	***	***	***
		Cultivar	42.97	10.85	0.628	*	***	***	*
SW	Spike weight (g)	Total	2.102	0.651	0.679	***	***	***	***
		Landrace	2.013	0.659	0.629	***	*	***	***
		Cultivar	2.284	0.594	0.597	**	*	***	*
PH	Plant height (cm)	Total	100.4	19.34	0.788	**	***	***	***
		Landrace	104.4	19.02	0.703	**	***	***	***
		Cultivar	92.03	17.22	0.788	**	***	***	***

*, ** and *** are significant at the probability level of 5%, 1% and 0.1%, respectively.

### Genetic data and population structure

**Figure 3:**
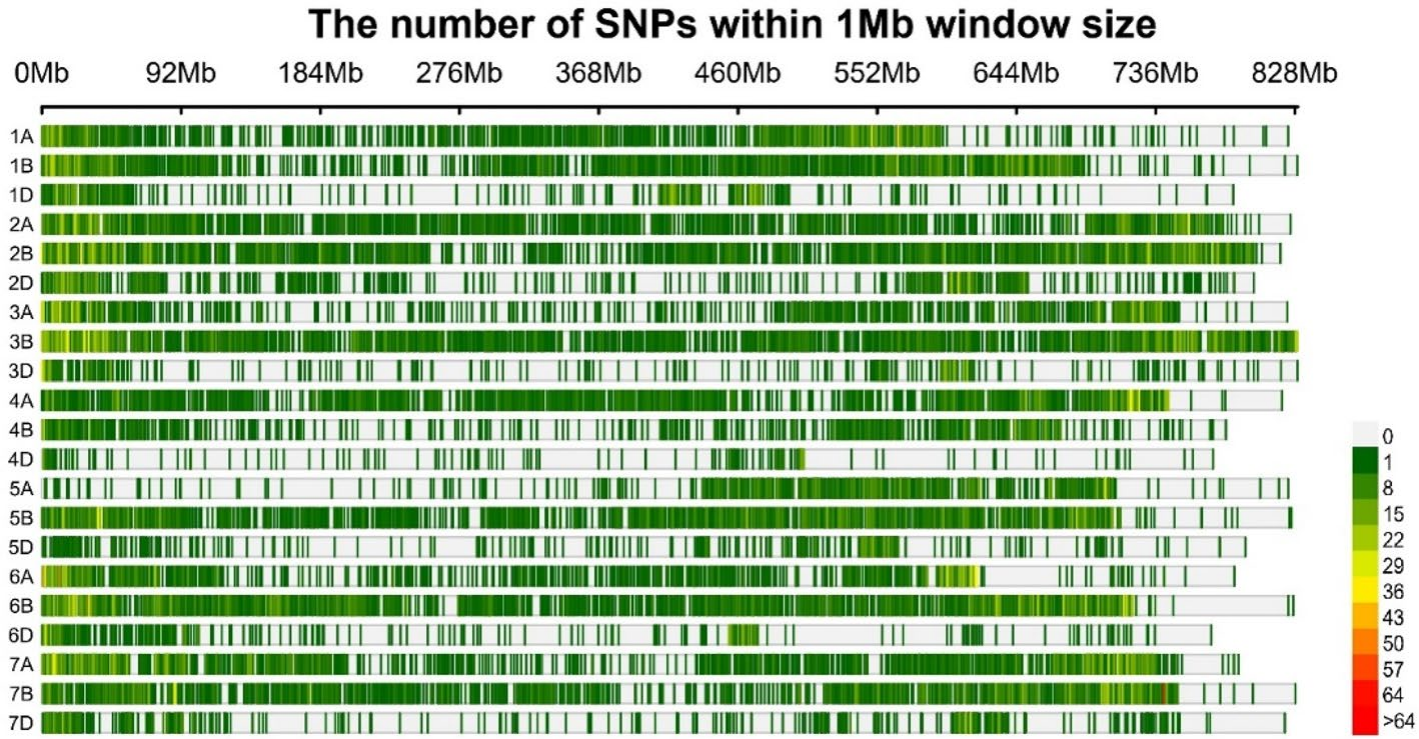
Density plot by different chromosomes in total Iranian bread wheat cultivars and landraces.

**Figure 4:**
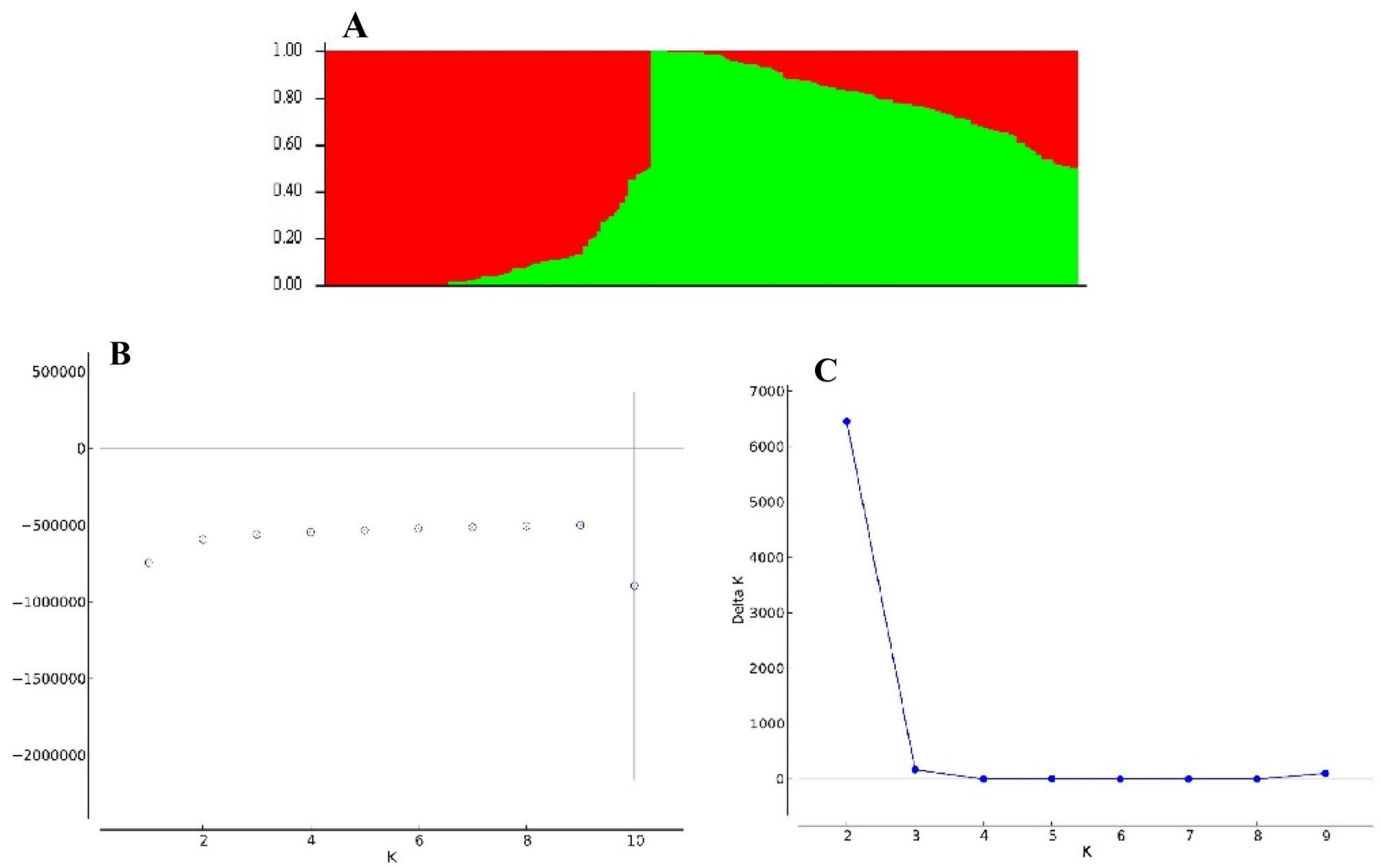
Barplot (**A**), the average log-likelihood value (**B**), and delta K for different numbers of sub-populations (**C**), in the analysis of population structure using 43,446 SNP markers.

Based on the results, the distribution of SNP markers showed that genome B alone accounted for 50% of the total markers, while genome D had the lowest number of SNP markers by far. In the A, B, and D genomes, chromosomes 7A, 3B, and 2D, respectively, had the highest number of SNPs in cultivars, landraces, and the sum of the two (Table 2). The density (SNP/Mbp) was similar, with the B genome having the highest density of SNPs, especially for chromosomes 6B and 3B. This is more conveniently illustrated in Fig. 3. The average minor allele frequency (MAF) and gene diversity (GD) in cultivars were slightly higher than the landraces. The amount of heterozygosity (HET) of the landraces in each of the chromosomes and consequently the genomes were higher than the cultivars. The polymorphism information content (PIC) in cultivars ranged from 0.240 (4D) to 0.309 (2A) and in landraces from 0.232 (2D) to 0.292 (4A). The mean PIC in cultivars, landraces, and the sum of these two was equal to 0.280, 0.267, and 0.270, respectively (Table 2). On the other hand, the total number of SNP pairs (TNSP) and the number of significant SNP pairs (NSSP) were higher in the B genome (especially on chromosomes 3B, 2B, and 6B) and lower in the D genome (especially on chromosomes 4D, 5D, and 3D). The percentage of NSSP in cultivars ranged from 25.11% (4D) to 58.26% (4A) and in landraces ranged from 26.16% (4B) to 53.27% (4A). The r^2^ values of cultivars were higher than landraces, especially in B and D genomes. Such a difference in distance (cM) can also be seen in the D genome (Table 3). The results of genetic population structure analysis indicated the existence of two subpopulations (Fig. 4A). The highest value of ΔK was observed at K = 2 (Fig. 4B), and its average log-likelihood value confirmed it (Fig. 4C). One of these subpopulations consisted mainly of cultivars, and the other contained landraces.

**Table 2:** Distribution of SNP markers and indices of genetic diversity by chromosomes.

Chromosome	Cultivar						Landrace					
	NS	D	MAF	GD	HET	PIC	NS	D	MAF	GD	HET	PIC
1A	1744	2.94	0.24	0.35	0.030	0.282	1985	3.34	0.20	0.31	0.033	0.252
1B	2640	3.83	0.26	0.36	0.026	0.287	2748	3.99	0.22	0.33	0.033	0.271
1D	777	1.57	0.21	0.32	0.024	0.258	852	1.72	0.22	0.32	0.032	0.261
2A	2419	3.10	0.30	0.39	0.031	0.309	2569	3.29	0.26	0.36	0.029	0.289
2B	3465	4.32	0.25	0.36	0.030	0.287	3168	3.95	0.24	0.35	0.033	0.281
2D	987	1.51	0.19	0.30	0.024	0.247	1170	1.80	0.17	0.28	0.028	0.232
3A	1746	2.33	0.24	0.34	0.032	0.278	1525	2.03	0.22	0.33	0.034	0.268
3B	3589	4.32	0.25	0.36	0.028	0.288	3547	4.27	0.20	0.32	0.030	0.260
3D	551	0.90	0.20	0.30	0.026	0.247	652	1.06	0.18	0.28	0.027	0.235
4A	2378	3.19	0.21	0.33	0.023	0.268	2265	3.04	0.29	0.37	0.026	0.292
4B	1288	1.91	0.21	0.32	0.023	0.257	924	1.37	0.21	0.31	0.029	0.252
4D	235	0.46	0.18	0.29	0.027	0.240	237	0.47	0.24	0.33	0.030	0.265
5A	1214	1.71	0.25	0.36	0.031	0.289	1211	1.71	0.21	0.32	0.034	0.264
5B	2754	3.86	0.26	0.37	0.030	0.292	2755	3.86	0.22	0.33	0.032	0.267
5D	474	0.84	0.22	0.33	0.027	0.271	534	0.94	0.18	0.29	0.029	0.240
6A	1737	2.81	0.24	0.35	0.026	0.281	1800	2.91	0.22	0.33	0.033	0.264
6B	3169	4.40	0.25	0.35	0.026	0.284	3375	4.68	0.23	0.34	0.032	0.275
6D	572	1.21	0.22	0.33	0.029	0.265	717	1.51	0.23	0.33	0.034	0.267
7A	2670	3.62	0.22	0.33	0.028	0.266	2616	3.55	0.24	0.34	0.032	0.277
7B	2757	3.67	0.24	0.34	0.031	0.276	2571	3.43	0.20	0.31	0.032	0.253
7D	731	1.14	0.20	0.30	0.027	0.249	859	1.35	0.20	0.31	0.030	0.249
Unknown	231	–	0.19	0.33	0.050	0.272	269	–	0.17	0.30	0.055	0.250
A genome	13,908	2.82	0.24	0.35	0.028	0.281	13,971	2.83	0.24	0.34	0.031	0.274
B genome	19,662	3.80	0.25	0.35	0.028	0.284	19,088	3.69	0.22	0.33	0.032	0.267
D genome	4327	1.10	0.21	0.31	0.026	0.254	5021	1.27	0.20	0.30	0.030	0.248
Whole genome	38,128	2.71	0.24	0.35	0.028	0.280	38,349	2.73	0.22	0.33	0.031	0.267

Chromosome	Total											
	NS	D	MAF	GD	HET	PIC						
1A	2233	3.76	0.21	0.31	0.030	0.256						
1B	2990	4.34	0.24	0.34	0.030	0.277						
1D	958	1.93	0.20	0.31	0.028	0.251						
2A	2715	3.48	0.28	0.38	0.030	0.298						
2B	3724	4.65	0.24	0.35	0.031	0.279						
2D	1352	2.07	0.16	0.27	0.027	0.228						
3A	1923	2.56	0.22	0.32	0.032	0.262						
3B	3996	4.81	0.24	0.35	0.029	0.282						
3D	722	1.17	0.17	0.27	0.025	0.229						
4A	2624	3.52	0.26	0.35	0.025	0.282						
4B	1202	1.78	0.20	0.30	0.027	0.245						
4D	267	0.52	0.21	0.31	0.028	0.255						
5A	1425	2.01	0.21	0.32	0.031	0.260						
5B	3027	4.25	0.25	0.35	0.031	0.284						
5D	634	1.12	0.18	0.28	0.026	0.236						
6A	1975	3.20	0.23	0.33	0.030	0.270						
6B	3770	5.23	0.24	0.34	0.029	0.277						
6D	755	1.59	0.21	0.32	0.031	0.259						
7A	2974	4.04	0.23	0.34	0.030	0.272						
7B	2985	3.98	0.21	0.32	0.031	0.258						
7D	911	1.43	0.20	0.30	0.029	0.249						
Unknown	284	–	0.17	0.30	0.055	0.251						
A genome	15,869	3.22	0.24	0.34	0.029	0.273						
B genome	21,694	4.19	0.23	0.34	0.030	0.275						
D genome	5599	1.42	0.19	0.29	0.028	0.242						
Whole genome	43,446	3.09	0.23	0.33	0.030	0.270						

*NS* number of SNPs, *D* density (SNP/Mbp), *MAF* minor allele frequency, *GD* gene diversity, *HET* heterozygosity, *PIC* polymorphism information content.

**Table 3:** A summary of observed LD (r^2^) among SNP pairs and the number of significant SNP pairs per chromosomes and genomes of Iranian bread wheat cultivars and landraces.

Chromosome	Cultivar				Landrace				Total			
	TNSP	r^2^	Distance (cM)	NSSP	TNSP	r^2^	Distance (cM)	NSSP	TNSP	r^2^	Distance (cM)	NSSP
1A	85,925	0.1484	1.7294	35,439 (41.24%)	97,975	0.1248	1.5165	33,986 (34.69%)	110,375	0.1142	1.3482	48,275 (43.74%)
2A	119,675	0.2887	0.9614	67,776 (56.63%)	127,175	0.2834	0.9147	65,687 (51.65%)	134,475	0.2573	0.8659	77,465 (57.61%)
3A	86,023	0.1587	2.5137	33,993 (39.52%)	74,975	0.1337	2.8859	26,776 (35.71%)	94,875	0.1341	2.2837	43,807 (46.17%)
4A	117,625	0.3719	1.4802	68,532 (58.26%)	111,975	0.3654	1.6121	59,648 (53.27%)	129,925	0.3217	1.3765	78,168 (60.16%)
5A	59,425	0.1685	2.3816	25,001 (42.07%)	59,275	0.1483	2.3842	22,256 (37.55%)	69,975	0.1364	2.0229	30,794 (44.01%)
6A	85,575	0.1799	1.4808	37,229 (43.5%)	88,725	0.1781	1.4283	38,821 (43.75%)	97,475	0.1621	1.3001	50,834 (52.15%)
7A	132,225	0.2269	1.3074	59,606 (45.08%)	129,525	0.2121	1.3348	61,524 (47.5%)	147,425	0.1978	1.1729	76,571 (51.94%)
1B	130,723	0.2081	1.0668	61,775 (47.26%)	136,125	0.1553	1.0221	61,072 (44.86%)	148,225	0.1595	0.9410	77,937 (52.58%)
2B	171,975	0.1960	0.8267	82,286 (47.85%)	157,125	0.1776	0.9048	74,524 (47.43%)	184,925	0.1614	0.7688	99,850 (53.99%)
3B	178,175	0.2431	0.8670	93,890 (52.7%)	176,075	0.2164	0.8775	86,960 (49.39%)	198,525	0.2152	0.7791	116,617 (58.74%)
4B	63,125	0.1958	2.0463	25,951 (41.11%)	44,925	0.1002	2.8745	11,754 (26.16%)	58,825	0.1196	2.2020	23,106 (39.28%)
5B	136,425	0.2029	1.4099	68,173 (49.97%)	136,475	0.1439	1.4305	54,163 (39.69%)	150,075	0.1544	1.3015	78,660 (52.41%)
6B	157,175	0.2086	0.7939	81,206 (51.67%)	167,475	0.1365	0.7451	67,171 (40.11%)	187,225	0.1415	0.6665	96,214 (51.39%)
7B	136,575	0.1550	1.0707	54,978 (40.25%)	127,275	0.1271	1.1430	46,545 (36.57%)	147,975	0.1248	0.9916	67,387 (45.54%)
1D	37,575	0.2956	4.3628	18,745 (49.89%)	41,325	0.2349	3.8014	19,089 (46.19%)	46,625	0.2489	3.5197	24,887 (53.38%)
2D	48,075	0.2330	2.2436	18,823 (39.15%)	57,225	0.1681	1.8916	20,518 (35.85%)	66,325	0.1918	1.6327	30,110 (45.4%)
3D	26,275	0.1386	6.0947	7046 (26.82%)	31,325	0.1738	5.1122	11,119 (35.5%)	34,825	0.1541	4.5999	13,102 (37.62%)
4D	10,475	0.1616	10.4006	2630 (25.11%)	10,575	0.1488	10.5114	3253 (30.76%)	12,075	0.1368	9.2176	4178 (34.6%)
5D	22,425	0.1544	9.3178	6748 (30.09%)	25,425	0.1371	8.2146	8542 (33.6%)	30,425	0.1377	6.9059	11,849 (38.94%)
6D	27,325	0.1348	5.5982	8589 (31.43%)	34,575	0.1406	4.4221	12,013 (34.74%)	36,475	0.1294	4.1966	14,920 (40.9%)
7D	35,275	0.2070	5.6652	13,201 (37.42%)	41,675	0.1476	4.8058	13,261 (31.82%)	44,275	0.1531	4.5205	17,051 (38.51%)
A genome	686,473	0.2332	1.5953	327,576 (47.72%)	689,625	0.2193	1.5990	308,698 (44.76%)	784,525	0.1992	1.4046	405,914 (51.74%)
B genome	974,173	0.2035	1.0559	468,259 (48.07%)	945,475	0.1590	1.0899	402,189 (42.54%)	1,075,775	0.1593	0.9600	559,771 (52.03%)
D genome	207,425	0.2029	5.3158	75,782 (36.53%)	242,125	0.1687	4.5376	87,795 (36.26%)	271,025	0.1735	4.0853	116,097 (42.84%)
Whole genome	1,868,071	0.2144	1.7271	871,617 (46.66%)	1,877,225	0.1824	1.7216	798,682 (42.55%)	2,131,325	0.1758	1.5211	1,081,782 (50.76%)

*TNSP* total number of SNP pairs, *NSSP* number of significant SNP pairs (*P* < 0.001).

### MTAs for mean traits and stability indices

**Figure 5:**
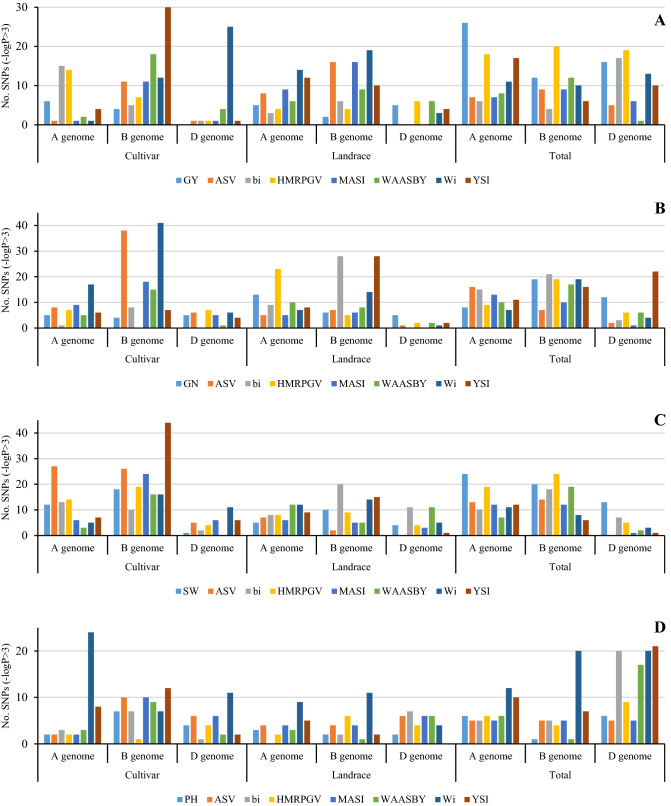
GWAS results stability indicators for grain yield (**A**), number of grains (**B**), spike weight (**C**), and plant height (**D**) in Iranian wheat landraces and cultivars.

**Figure 6:**
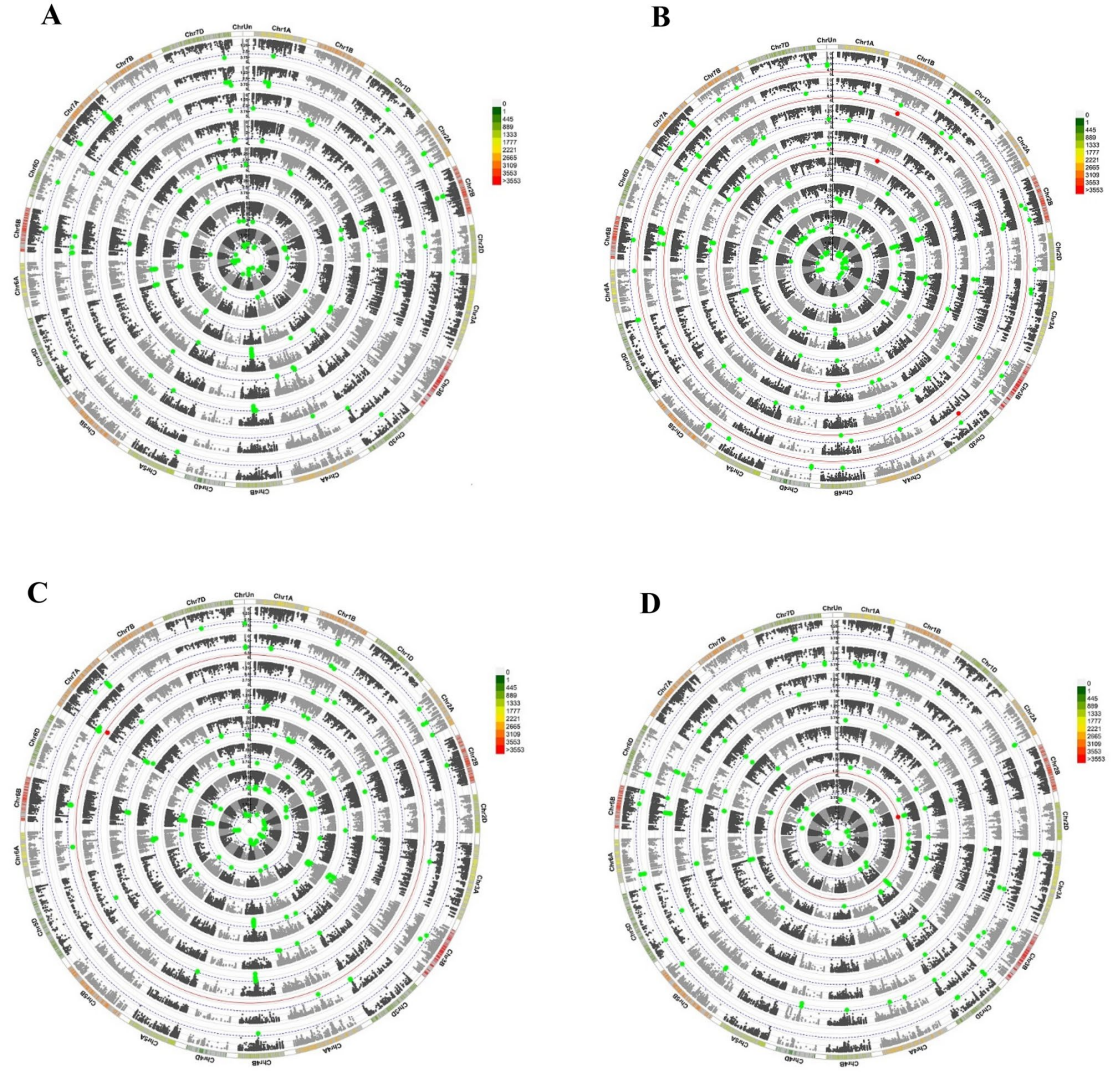
Circular Manhattan plots to draw common regions associated with grain yield (**A**), number of grains (**B**), spike weight (**C**), and plant height (**D**) in Iranian wheat landraces and cultivars. Inner to outer circles represents average trait and stability indices including ASV, bi, HMRPGV, MASI WAASBY, Wi, and YSI, respectively. The chromosomes are plotted at the outmost circle where thin dotted blue and red lines indicate significant level at *p* value < 0.001 (− log_10_ (*p*) > 3) and < 0.00001 (− log_10_ (*p*) > 5), respectively. Green and red dots indicate genome-wide significantly associated SNPs at *p* value < 0.001 and < 0.00001 probability level, respectively. Scale between ChrUn and Chr1A indicates − log_10_ (*p*) values. Colored boxes outside on the top right side indicate SNP density across the genome where green to red indicates less dense to dense.

An overview and detailed information of MTAs results are provided in Supplementary Tables 4 and 5. A total of 846, 653, and 1023 significant MTAs were identified for the studied traits and stability indices of cultivars, landraces, and total genotypes, respectively (Fig. 5). Circular Manhattan plots for common regions associated with different traits are plotted (Fig. 6). Ten and 12 markers were related to the mean grain yield of cultivars and landraces, respectively, mainly located in genome A. This number was higher with 55 markers for all genotypes. ASV and MASI statistics had the highest MTAs in the B genome, while for W_i_ in the D genome (especially chromosome 7D) and the B genome, most markers in landraces were identified on the B and A genomes. There were 22 and 14 significant associations for HMRPGV in cultivars and landraces, respectively. Chromosomes 4A and 2A for cultivars and 3D for landraces were important. WAASBY was significantly associated with 24 and 21 SNPs in cultivars and landraces. These markers were mainly distributed on chromosomes 6B, 2B, and 2D. Although b_i_ for cultivars and landraces had the lowest MTAs in the D genome, this genome (especially its 6D chromosome) contained the highest MTAs considering the total genotypes. Finally, among all the indices, YSI in the cultivars was associated with the highest number of SNPs in the B genome (Fig. 5A).

For GN, 14 MTAs for the mean and 209 MTAs for the stability parameters were identified in the cultivars, compared to 24 and 171 MTAs for the landraces, respectively. Like GY, more MTAs were identified based on all genotypes. Chromosomes 6B and 2B in cultivars contained the highest markers associated with ASV and MASI, while the SNPs identified for these two indices were low in landraces and scattered on different chromosomes. Genome B, especially chromosome 2B, had the highest QTLs associated with W_i_. In total, 14 and 30 MTAs were determined for HMRPGV in cultivars and landraces, respectively, with chromosomes 1A, 4A, and 5A having the highest SNPs in the landraces. The highest number of SNPs associated with WAASBY in cultivars and landraces were located in B and A genome, respectively. The highest number of b_i_ and YSI-related SNPs belonged to the B genome, and chromosomes 6B and 7A in landraces were important for the YSI index (Fig. 5B).

Mean SW in cultivars, landraces, and total genotypes was identified as 31, 19, and 57 MTAs, respectively, mainly located on chromosomes 6A, 3B, 4B, 6B, and 7D. The B genome and then the A genome had a highly significant number of HMRPGV-related SNPs. For WAASBY in the cultivars, 16 and 3 MTAs were identified in the B and A genome, respectively. Although no significant MTAs were observed in genome D cultivars, like the A genome, it contained SNPs related to the WAASBY index in the landraces. In terms of ASV and MASI indices, 6.4 and 2.6 times more MTAs were detected in the cultivars compared to landraces, respectively. More MTAs were observed on chromosomes 6B and 6D in cultivars and 1B, 3D, 6A, 6B, and 7B in landraces for W_i_ index. Most b_i_-related SNPs were located on chromosomes 1A, 6B, and 7A in the cultivars and on 1D, 3B, 6B, and 7A in the landraces. Finally, for the SW, like GY, the highest number of MTAs we could see in a genome was the YSI index (Fig. 5C).

Among the traits, the lowest MTAs were observed for PH and its stability indices. Moreover, 13 markers on 1D, 2B, 3B, 5B, 7A, 7B, and 7D chromosomes in cultivars and seven markers on 1A, 1D, 2A, 5B, and 7B chromosomes were associated with mean trait. The markers identified for ASV and MASI were the same in the cultivars and slightly different in the landraces. Such similarity was observed by considering the sum of genotypes, with 3D and 7A chromosomes having a larger number of SNPs. Although genome B had the lowest number of MTAs for W_i_ in cultivars, it showed the highest association in landraces. Chromosomes 3A, 6D, and 7A in cultivars and chromosomes 4A and 6B in landraces were important for this index. For HMRPGV in cultivars, seven markers were identified on chromosomes 6D, 7A, 3D, and 5B. These numbers were equal to 12 and were distributed on chromosomes 7B, 5D, 5B, 1D, 1A, 6A, and 2B. According to WAASBY, 14 SNPs were identified in cultivars on different chromosomes, including 1A, 1B, 2B, 3B, 3D, 4B, 4D, 5B, 6A, and 7B. In the landraces, 10 SNPs were identified, more than half of which were located on chromosome 1D. The b_i_, in cultivars on chromosomes 6B and in the landraces on chromosomes 3D and 3D, had the highest number of MTAs. Finally, the number of MTAs detected for YSI in cultivars was three times higher than in landraces (Fig. 5D).

Among the identified markers, 171, 131, and 224 cases in cultivars, landraces, and the sum of these two overlapped with different traits and indices, respectively (Supplementary Table 5). For example, the marker rs65138 in cultivars and rs51479 in total genotypes were associated with the mean of three traits GY, GN, SW, and some of their stability indices and were located on chromosomes 1B and 3B, respectively. One such marker in the landraces was rs58587, which was located on chromosome 7B and was associated only with the stability indices of GY, SW, and PH. Other SNPs with many pleiotropy effects were located on chromosomes 6B, 2A, 2B, 4D, 3B, and 4A in the cultivars. These cases in the landraces included 1A, 2A, 4A, 2D, 6A, 3D, 1B, and 7D. Considering the total genotypes, we found that the SNPs associated with most of the traits and indices were on chromosomes 4B, 4A, 2A, 2B, 7D, 2B, 6A, and 5D (Supplementary Table 5).

### Gene ontology

For a closer look, we studied the ontology of highly significant markers (*P* < 0.0001). Except for PH, some of the identified MTAs were involved in important biological and molecular processes for all traits. These genes were distributed on different chromosomes, including 1A, 1B, 1D, 2D, 3A, 4A, 4B, 6A, 6B, and 7A, with chromosome 4B, 1B, and 7A having the highest number (Table 4). Genes with MTAs mainly encoded proteins wrapped in biological and molecular processes associated with adaptation, including drought stress tolerance. Oxidoreductase activity, DNA-binding transcription factor activity, ATPase-coupled transmembrane transporter activity, protein kinase activity, protein binding, and integral component of the membrane were some of the molecular processes. Some biological processes also included the oxidation–reduction process, regulation of transcription, jasmonic acid biosynthetic process, transmembrane transport, protein phosphorylation, fatty acid biosynthetic process, and DNA repair. The KEGG orthology system was also used to accurately annotate the identified SNPs. The results showed that genes were involved in various pathways such as biosynthesis of secondary metabolites, carotenoid biosynthesis, fatty acid elongation and ubiquinone, and another terpenoid-quinone biosynthesis (Table 5).

**Table 4:** Description and annotation of identified markers (*P* < 0.0001).

No	SNP	Sequence	Trait- Index	Chromosome	Position (bp)	Molecular process	Biological process
1	rs10875	TGCAGCAAAGAAAGGAGAGCAC GAGGGGGTGGCCCAGGCCCTTA CCGTGAACAGCTCGCCGAGA_20	SW- Mean	4B	56.065	Oxidoreductase activity	Oxidation–reduction process
2	rs2321	TGCAGAAGTAGAACCAGAGGCG CCCTTCTCCTCTCTATACCCCGC AACCGTCACAGGATTAATA_48	GY- Mean	6A	55.893	DNA-binding transcription factor activity	Regulation of transcription, DNA-templated
3	rs30755	TGCAGCGCGGCAACGCCGTGGT CGTCATGTCGGGCTTCGCCATG GACTCCGTGATGAGGGCCGT_13	SW- WAASBY	4B	58.338	Fatty-acyl-CoA synthase activity	jasmonic acid biosynthetic process
4	rs41275	TGCAGCTTGATCACGCGCATGT AGCTGAGCAACTCGGTGATGGC CTTCATGCGCTCGTCTCGCT_43	GN- Wi	NA	NA	ATPase-coupled transmembrane transporter activity	Transmembrane transport
5	rs41740	TGCAGCTTTAACACTGTTTAACC CCCCCTGTGCAGCCTGATGGCC AGATGCCCGAGATCGGAAG_21	GN- Wi	1D	77.324	Structural constituent of cytoskeleton	Microtubule cytoskeleton organization
6	rs53737	TGCAGGTGAGCCGCCGAGCTGC TGCTGCTGCTTCCGCCCGATTT GATTTACAAATTCTGTTCTG_37	SW- YSI SW- ASV	1B	104.719	Anaphase-promoting complex	-
7	rs57405	TGCAGTATCTGAGTGTGAACTA GTCGCAGTGACAATGCATGTCG TTAAAAAGAATATGAACTAC_55	SW- YSI GY- YSI	7A	111.704	Protein kinase activity	Protein phosphorylation
8	rs57539	TGCAGTATTCATAGTGTGGCTT TGAGTGGAACTACACGATTTAG AGTTCACCACCTGCATTCTG_31	SW- YSI	6B	50.104	Peptidase activity	Signal peptide processing
9	rs5823	TGCAGAGCACGAAGTCCACGG CGTGATCCTTTTACTTTATTCCT TAAGCCAAGGGAGGTCGTAC_18	GY- Mean	4A	9.109	Oxidoreductase activity	Oxidation–reduction process
10	rs59777	TGCAGTCTTTCAGAAGTGCAGA TGTAAACGTATTGCTATATCAG TGGTTTGAACTACATGGTAA_10	GY- WAASBY	2D	58.883	Protein binding	Positive regulation of protein catabolic process
11	rs63903	TGCAGTTGAGGACAAGCACACG GATGGAGTCTGGGGCGACGCCT GTCCTGGAGAGCAGGTCATC_13	GY- YSI	7A	111.704	Transferase activity, transferring acyl groups other than amino-acyl groups	Fatty acid biosynthetic process
12	rs6859	TGCAGAGGGCGCGCGGGGACAG AGTGAATCGGGCAGAAGCAGAG GAGGATAAGAGAGACGAAGC_16	GN- bi	1B	66.042	Integral component of membrane	–
13	rs6887	TGCAGAGGGGGGCCAGGTAGGC GTGTGCTATGGGAGGATGGCCA CTAACCTGCCTGACCCGACG_42	SW- bi	1A	111.964	Hydrolase activity, hydrolyzing O-glycosyl compounds	–
14	rs736	TGCAGAAAGGTACCACTCATTC GTACATCACTCCAACTGATGTA TGAAGGTTGTTCATGGCGAC_18	SW- HMRPGV	4B	56.065	Hydrolase activity	Phosphatidylinositol dephosphorylation
15	rs2302	TGCAGAAGTAAAGAAGCTGAGA TGCGAGACAGTATAAATTTGCT AATAGACTAGCTTTGAAAGA_28	GN- WAASBY	3A	11.391	–	DNA repair

*GY* grain yield, *SW* spike weight, *GN* grain number.

**Table 5:** KEGG orthology-based annotation system for significant SNP sequences.

Term	ID	Chromosome	Pathway
Biosynthesis of secondary metabolites—unclassified	osa00999	IWGSC:4B:544042415:544043078:-1	Not found
Carotenoid biosynthesis	osa00906	IWGSC:4A:3139025:3139688:1	Supplementary Fig. 2
Fatty acid elongation	osa00062	IWGSC:7A:711171746:711172409:1	Supplementary Fig. 3
Ubiquinone and other terpenoid-quinone biosynthesis	osa00130	IWGSC:4B:544042415:544043078:-1	Supplementary Fig. 4

## Discussion

The high significance of GEI for the studied traits was expected in this study, which accords with the previous reports^9^. Similar to this study, different monthly rainfall in MET studies, which also has drought stress, is one of the main reasons for GEI^10,33^. For some traits, the effect of GEI in cultivars was less than in landraces. This result is due to breeding programs and the small number of samples in the cultivars compared to the landraces, leading to fewer effects of GEI. Severe GEI caused low heritability of traits, especially in GY. In general, heritability and repeatability for complex traits such as GY are low compared to PH^9,28,34^. High yield and stability of wheat cultivars were expected since new wheat genotypes tolerate adverse environmental conditions such as drought stress^35,36^. On the other hand, breeding programs have improved wheat adaptation throughout a century^25^ and continued to provide adapted wheat germplasm^37^. The genotypes in the fourth group in each of the traits, which included unstable low-yield genotypes, mainly consisted of landraces. Also, landraces had the highest percentage of genotypes selected by the multi-trait stability index. Most likely, the lack of specific selection for high yield and priority of yield stability during wheat domestication has led to such a result^38^. However, severe genetic heterogeneity in the Iranian wheat landraces and the application of some early breeding processes^39^ have put landraces in desirable groups in terms of yield and stability. In this context, additional assessments with a large number of locations are needed to fully explain GEI patterns.

The concepts of static and dynamic stability can be clearly distinguished based on bi and Wi indices in GY. The genotypes of group I, i.e., most cultivars, had dynamic stability. In contrast, although unstable in terms of dynamic concept, the fourth group, including the landraces, had static stability due to the low values for bi. The static concept is associated with low GY^8^. The genotypes of the second group, which included a small number of cultivars and landraces, were unstable in terms of both concepts despite their high yield and had good adaptability according to WAASBY and HMRPGV indices. We found a distinction between the concepts of stability and adaptability in other traits, especially PH. However, the literature paid scant attention to such a distinction between cultivars and landraces in terms of stability.

We found that the studied SNPs covered the wheat genome well. The number of SNPs based on the new wheat reference genome was higher in the B genome and lower in the D genome. There also seemed to be a direct relationship between marker density and chromosome size, and such a frequency of SNPs results from the evolutionary process of wheat. This conclusion was reported by Alipour et al*.*^39^ in Chinese Spring and W7984 reference genomes. Other similar results were confirmed by Mourad et al*.*^40^ and Edae et al*.*^41^. The difference of r^2^ in cultivars, landraces, and different chromosomes, in addition to the evolutionary process, indicates the effect of breeding programs^42^. In this regard, comparing landraces and cultivars of wheat in China and Pakistan showed that the distances of LD decays in the landraces were less than cultivars. On the other hand, LD decays in genome A was slower than that of B^43^. Given that the landraces are genetically heterogeneous and are collected from areas with different climates, we expected that their heterozygosity would be high. Environmental factors affect genetic diversity and the structure pattern of plant populations^44^. Therefore, the high level of gene diversity in the studied population can be attributed to the geographical diversity of collection sites, differences in growth habit, etc. These factors led us to observe two subpopulations that separated cultivars well from the landraces. Moreover, the breeding programs and improved accessions are the reasons for such a separation. Iranian wheat genotypes have been categorized into two subpopulations in the previous studies^40^. The mean PIC value for all genotypes was 0.27, which is a good value for the bi-allelic marker^39,45^, and given their good distribution throughout the genome, they can be used to understand the genetic basis of GEI control.

Genome-wide association studies capture the genetic loci linked to significant variation for traits of interest in a vast collection of wild relative populations, breeding cultivars, and landraces^46,47^. It is also an important tool for selecting high-yield genotypes in a group of environments^33^. In the current study, genomic regions controlling GY, GN, SW, and PH traits and stability indices based on these traits were identified on all 21 chromosomes, including those that were not mapped to any chromosome. The number of MTAs identified in all genotypes was higher for GY and its indices in the D genome compared to the B genome, and for PH, it was higher than in the A and B genomes. In a study, GWAS for GY was run in each environment due to the presence of GEI, and the results showed that the D genome had the highest number of SNPs^33^. This suggests that the role of the D genome in wheat adaptability should be further addressed^48^. The greatest number of significant MTAs were identified on chromosome 6B in both cultivars and landraces datasets, while the least numbers were detected on chromosomes 5D and 4D in cultivars and landraces datasets, respectively. Acuña-Galindo et al*.*^49^ also found two meta-QTL for adaptation to drought stress on chromosome 6B in wheat. A recent study also reported a major grain yield QTL on chromosome 6B and fifteen haplotype blocks associated with two stability indices, including Lin and Binn’s superiority index and Eberhart and Russell’s coefficient on chromosomes 1A, 4A, 4B, 5B, 6B, 7A, 7B, and 7D^29^. In addition, genomic regions associated with grain yield and yield stability on chromosomes 2B, 3A, 4A, 5B, 7A, and 7B were identified in CIMMYT’s spring bread wheat^50^. Considering all genotypes, we located about 44% and 24% of the markers associated with the mean GY on chromosomes 6A and 7D, respectively.

Interestingly, GO results showed that one of these markers is in the coding region of proteins that regulate transcription. Previous reports indicate that chromosome 6A contains GY and TGW-related locus in MET data that harbored a TaGW2-6A gene and that other genes influence its expression^51^. Chromosome 7D is of great importance in explaining GY phenotypic variation^28^. Muhu-Din Ahmed et al*.*^52^ identified MTAs for GY on chromosomes 1A, 3A, 4A, 1B, 4B, 6B, 7B, 5D, and 7D under both well-watered and water-deficit conditions. Several studies also demonstrated MTAs for GY in various wheat panels analyzed thorough GWAS on chromosomes 2B, 3A, 3D, 5B, 7A and 7B^53^, 1A, 2D, 3A, 7B and 7D^1^, and 1B^54^ under different water regimes. The marker locus on 4B in GY under water stress conditions was also associated with this trait in the Pakistani wheat population^55^. Similarly, in genome-wide association mapping, Edae et al.^56^ reported MTAs for GY on chromosomes 4A, 1B, 5B, and 2B of spring wheat association panel under contrasting moisture regimes. Moreover, Lozada et al*.*^57^ found MTAs for GY on chromosomes 5A, 1B, 2B, and 4B in a diverse panel of 239 wheat genotypes evaluated across two growing seasons using SNP markers. Tadesse et al*.*^58^ reported GY-related MTAs on 1B in 120 elite hexaploid wheat genotypes, which were evaluated under rain-fed and irrigated conditions for a genome-wide study.

The multi-trait loci controlling performance and stability were located on chromosomes 1B, 3B, and 7B. Furthermore, chromosomes 2A and 4A in all three cultivars, landraces, and the sum of these two had multi-trait control loci. All chromosomes, except for chromosome 3B, were reported in a similar study^9^. In another study, chromosomes 3B and 2B, 3A, 4A, 5B, 7A, and 7B were associated with wheat yield stability coefficient^50^. Major QTLs with pleiotropic effects on chromosomes 3B and 7B have also been confirmed^59^. One study concluded that a specific combination of photoperiod genes increases the yield stability of durum wheat^14^. Also, the best allelic combination using stepwise regression in markers identified by genome-wide association mapping (GWAM) can lead to increased stability and yield in wheat^50^. Therefore, it is possible to say that yield stability is controlled by genes with pleiotropic effects. However, as the experiment was performed in the same place and under different conditions, the correlation between grain yield in different environments may be a reason to observe common SNPs. In this regard, the lack of correlation between the environments resulted in no common SNP for the GWAS performed in 9 environments^33^. Although several common MTAs were identified in for GY, GN, SW, and PH traits and different stability indices, these traits are not exclusive and independent. Thus, it is possible to select both traits and stability indices in Iranian wheat cultivars and landraces since most significant MTAs (almost 90%) were not common among the trait values and stability indices. Lozada and Carter^9^ identified 12 SNP loci linked to both trait value and stability parameters in Pacific Northwest winter wheat. Two major effect SNP markers of *Tdurum_contig61410_542* (1B) and *BS00022542_51* (7B), were associated with grain yield and yield stability indices. The common MTAs between different traits and yield stability coefficient have already been reported^50^. The low number of MTAs identified for PH is probably due to the fact that this trait is controlled by a small number of genes compared to other traits. However, the above results for yield and its components show that they are controlled by several genes that interact with each other and the environment. Stability-associated genes can also be stress-responsive genes^50^. Therefore, GO results could be well described, given that two of the six environments are under rain-fed conditions. Proteins phosphorylation, especially in wheat grains, play an important role in drought stress^60^. Jasmonic acid biosynthetic modulates drought stress in wheat^61^. Markers related to mean GY and SW were annotated with antioxidant activity. Reducing the effects of drought stress by such activity with various enzymes in wheat was demonstrated by previous researchers^62^. The Synthesis of fatty acids is useful in counteracting the drought stress in oats^63^. Transmembrane transport, DNA-binding transcription factor activity, DNA repair, and peptidase activity were other examples that were annotated and possibly involved in response to drought stress. These results are similar to the previous reports^64^. Earlier efforts have been made to interpret GWAS results and understand GEI using gene annotation^33^. KOBAS is a useful tool for genome annotation^65^. It has been shown that ubiquinone and other terpenoid-quinone biosynthesis are metabolic pathways of response to drought stress in plants^66^. In addition, carotenoid biosynthesis is involved as one of the KEGG pathways in drought stress tolerance^67^. Such an important role for the biosynthesis of secondary metabolites has been proven^68^.

## Conclusions

In the current study, GWAS was performed for some important agronomic traits and different static and dynamic stability indices based on those traits were calculated in a diverse panel of 268 Iranian wheat cultivars and landraces. The highest number of marker pairs and lowest LD decay distance in both cultivars and landraces was observed on the B genome, whereas the D genome had the least number of marker pairs and most significant LD decay distance. A total of 846, 653, and 1023 significant MTAs were identified for the traits and their related stability indices in cultivars, landraces, and total genotypes datasets, respectively. The chromosomes 6B and 4D had the highest and lowest number of MTAs, respectively. The multi-trait loci controlling mean traits and stability were located on chromosomes 1B, 3B, and 7B, and GO results for highly significant MTAs almost confirmed the accuracy of the identified markers. The identified markers in this study could provide valuable genetic resources to initiate marker-assisted selection, fine mapping, and cloning the underlying genes and QTLs.

## Methods

### Plant materials and field evaluation

A set of 268 Iranian bread wheat genotypes, including 180 landraces and 88 cultivars, were studied in six environments (Supplementary Table 1). The environments included four well-watered environments during 2014, 2015, 2017, and 2018 and two rain-fed environments in 2017 and 2018 (Supplementary Table 2). Trials were planted in early November and harvested in July of the next year. The experiments were performed on the research farm of the University of Tehran with latitudes of 50.58 E and 35.56 N and 1112.5 m above sea level in a randomized complete block design with two replications. The dimensions of the plots consisted of four lines with a length of 1 m (80 × 100 cm). The distance was 20 and 5 cm between and within the rows. Plant height (PH, cm), grain number per spike (GN), spike weight (SW, g), grain yield per plant (GY, g plant^-1^) were traits that were measured based on ten randomly selected samples from each plot. Plant height was recorded from ground level to tip of the spike, excluding awns, at maturity stage. After harvesting, all spikes were hand-threshed to determine the GY, SW, and GN. Then, stability parameters (Table 6) of each trait were calculated using ‘agricolae’^69^, ‘ammistability’^18^, and ‘metan’^70^ packages in the R and STABILITYSOFT online programs^71^. Broad sense heritability of traits was calculated using the following equation:

$$ H^{2} = \sigma_{g}^{2} /(\sigma_{g}^{2} + (\sigma_{ge}^{2} /e) + (\sigma_{\varepsilon }^{2} /er)) $$

where $\sigma_{g}^{2}$ and $\sigma_{ge}^{2}$ are the variance due to genotype, and genotype-by-environment interaction, respectively. $\sigma_{\varepsilon }^{2}$ is the residual variance, and e and r are the number of environments and replications, respectively^72^.

**Table 6:** Description of the stability statistics studied.

Stability measures	Details	References
Wricke’s ecovalance measures	$W_{i} = \sum \left( {X_{ij} - \overline{X}_{i \cdot } - \overline{X}_{ \cdot j} + \overline{X}_{ \cdot \cdot } } \right)^{2}$	^16^
Finlay-Wilkinson regression	$b_{i} = \frac{{\mathop \sum \nolimits_{j} X_{ij} I_{j} }}{{\mathop \sum \nolimits_{j} I_{j}^{2} }}$	^19^
AMMI stability value	$ASV = \sqrt {\left( {\frac{{SSPC_{1} }}{{SSPC_{2} }} \times PC_{1} } \right)^{2} + \left( {PC_{2} } \right)^{2} }$	^17^
Harmonic mean of the relative performance of the genetic values	$HMRPGV = \left( {\frac{1}{{\mathop \sum \nolimits_{e = 1}^{e} \frac{1}{{RPGV_{ij} }}}}} \right)$; $RPGV = \frac{1}{e}\left( {\frac{{\sum GV_{ij} }}{{\mu_{j} }}} \right)$ $GV_{ij} = u_{j} + g_{i} + ge_{ij}$	^21^
Modified AMMI stability index	$MASI = \sqrt {\mathop \sum \limits_{n = 1}^{{\mathop N\limits^{{\prime }} }} PC_{n}^{2} + \theta_{n}^{2} }$	^18^
Yield stability index	$YSI = RASV + RY$	^22^
Weighted average of absolute scores from the singular value decomposition of the matrix of BLUP for the GEI effects generated by an LMM and response variable	$WAASBY = \frac{{\left( {rG_{g} \times \theta_{Y} } \right) + \left( {rW_{g} \times \theta_{S} } \right)}}{{\theta_{Y} + \theta_{S} }}$ $rG_{g} = \frac{100 - 0}{{G_{max} - G_{min} }} \times \left( {G_{g} - G_{max} } \right) + 100$ $rW_{g} = \frac{0 - 100}{{W_{max} - W_{min} }} \times \left( {W_{g} - W_{max} } \right) + 0$ $WAASB = \mathop \sum \limits_{n = 1}^{p} \left| {IPCA_{gn} \times EP_{n} } \right|/\mathop \sum \limits_{n = 1}^{p} EP_{n}$	^23^

*g*: number of genotypes, *e*: number of environments, *X*_*ij*_: average yield of genotype *i* in environment *j*, ${\overline{X} }_{i \cdot }$: average yield of the genotype *i*, ${\overline{X} }_{\cdot j}$: average yield of the environment *j*, ${\overline{X} }_{\cdot \cdot }$: grand average yield, *Ij*: Environmental index, which is the deviation of the average of all genotypes in a specific place from the total average, *PC*: interaction principal components, *SSPC*_*n*_: sum of squares of the *n*th PC, *GV*_*ij*_: genotypic value of genotype *i* in environment *j*, *u*_*j*_: represents the mean of environment *j*, *g*_*i*_ and *ge*_*ij*_ are the BLUP values of genotype *i* and the interaction between genotype *i* and or environment *j*, respectively, *θ*_*n*_: the percentage sum of squares explained by the nth principal component interaction effect, *RASV*: rank of AMMI stability value, *RY*: rank of the mean yield of genotypes across environments, *μ*_*j*_: general mean for each environment *j*, *IPCA*_*gn*_ is the score of the genotype *g* in the *n*th IPCA, and *EP*_*n*_ is the amount of the variance explained by the *n*th IPCA. *θ*_*Y*_ and *θ*_*S*_ are the weights for yield and stability, respectively. *rG*_*g*_ and *rW*_*g*_ are the rescaled values of the *g*th genotype for yield and WAASB, respectively, *G*_*g*_ and *W*_*g*_ are the yield and WAASB values for *g*th genotype, respectively.

### Genotyping

The development and genetic material studied was previously described based on genotyping by sequencing of a GBS library for the Iranian wheat samples have been by Alipour et al.^39^. In brief, sequence reads were first trimmed to 64 bp and were grouped into sequence tags. Then, SNPs were identified using internal alignment allowing for mismatch up to 3 bp. The UNEAK (Universal Network-Enabled Analysis Kit) GBS pipeline was used for SNPs calling, where reads with a low-quality score (< 15) were discarded. Imputation was performed in BEAGLE v3.3.2^73^ using w7984 reference genome^74^. Finally, SNPs with heterozygotes < 10%, and minor allele frequency > 5% were used for further analysis.

### Genome-wide association study

Both general linear model (GLM) and mixed linear model (MLM) were employed to obtain the unbiased estimation of marker effects using TASSEL 5.0^75^ software and GAPIT R-package^76^. The results of GLM was adjusted using the first three principal components (PCA) and population structure (Q) and MLM was corrected using kinship-matrix with the first three principal components (PCA + K) and population structure (Q + K). Results of all approaches from both TASSEL and GAPIT were evaluated based on the Q-Q plot and significance of associated loci using t-tests. In general, the results of the MLM approach of the first three principal components and kinship-matrix (PCA + K) obtained from GAPIT provided a more robust control of confounding effects. We, therefore, only reported the results MLM obtained from GAPIT. In the MLM model, individuals are considered random effects, and the relatedness among individuals is conveyed through a kinship matrix. A threshold of –log_10_ (*p*) > 3 was used to state statistically significant MTAs^77,78^. Confidence intervals (CIs) for MTAs were calculated for each chromosome using the linkage disequilibrium (LD) decay. Circular Manhattan plots were performed using the CMplot R-package^79^.

### Gene annotation

Sequences surrounding all significantly associated SNPs were obtained from the blast tools in EnsemblPlants database (http://plants.ensembl.org/index.html) to assess gene annotation using Gramene (http://www.gramene.org/) by aligning them to the IWGSC RefSeq v1.0 annotation (https://wheat-urgi.versailles.inra.fr/Seq-Repository/Annotations). After aligning SNPs sequences to the reference genome, we selected overlapping genes with the highest identity percentage and blast score for further processing. The gene ontology of each selected gene, including molecular function and biological process, was extracted from the ensemble-gramene database (http://ensembl.gramene.org). In addition, the sequences of significant SNPs were used for GO enrichment analyses using KOBAS (KEGG Orthology-Based Annotation System) software^80^ to test for statistically enriched pathways in the Kyoto Encyclopedia of Genes and Genomes (KEGG, https://www.genome.jp/kegg/) database.

## Supplementary Information

Supplementary Information 1.

Supplementary Table 4.

Supplementary Table 5.
