# Supplementary material for: Dissection of the genetic basis of genotype-by-environment interactions for grain yield and main agronomic traits in Iranian bread wheat landraces and cultivars
Source: Sci Rep. 2021 Sep 7;11:17742. doi: 10.1038/s41598-021-96576-1 (PMC8423731; doi:10.1038/s41598-021-96576-1)
Supplement: Supplementary file 1 — Supplementary Information 1. [file 41598_2021_96576_MOESM1_ESM.docx]

**Supplementary Table 1.** Overview on the landraces and cultivars of Iranian wheat studied

| Genetic background: Landraces | | | | | | | | | |
| --- | --- | --- | --- | --- | --- | --- | --- | --- | --- |
| No. | USDA_PI_NO | | Region of origin (Province) | |  | No. | USDA_PI_NO | | Region of origin (Province) |
| 1 | 621421 | | Hamadan | |  | 91 | 625080 | | Markazi |
| 2 | 621492 | | Bakhtaran | |  | 92 | 625081 | | Markazi |
| 3 | 621565 | | Hamadan | |  | 93 | 625123 | | Markazi |
| 4 | 621619 | | Kordestan | |  | 94 | 625127 | | Markazi |
| 5 | 621650 | | Tehran | |  | 95 | 625281 | | Gilan |
| 6 | 621668 | | Tehran | |  | 96 | 625362 | | Mazandaran |
| 7 | 621669 | | Tehran | |  | 97 | 625433 | | Khorasan |
| 8 | 621704 | | Gazvin | |  | 98 | 625661 | | Khorasan |
| 9 | 621706 | | Gazvin | |  | 99 | 625810 | | Khorasan |
| 10 | 621735 | | Gazvin | |  | 100 | 626156 | | Kerman |
| 11 | 621908 | | Markazi | |  | 101 | 626158 | | Kerman |
| 12 | 622063 | | Zanjan | |  | 102 | 626215 | | Kerman |
| 13 | 622084 | | Mazandaran | |  | 103 | 626223 | | Sistan-Balouchestan |
| 14 | 622247 | | Mazandaran | |  | 104 | 626226 | | Sistan-Balouchestan |
| 15 | 622311 | | Khorasan | |  | 105 | 626234 | | Sistan-Balouchestan |
| 16 | 622379 | | Khorasan | |  | 106 | 626260 | | Sistan-Balouchestan |
| 17 | 623008 | | Esfahan | |  | 107 | 626261 | | Sistan-Balouchestan |
| 18 | 623069 | | Esfahan | |  | 108 | 626358 | | Esfahan |
| 19 | 623090 | | Bakhtaran | |  | 109 | 626360 | | Esfahan |
| 20 | 623091 | | Khorasan | |  | 110 | 626565 | | Esfahan |
| 21 | 623109 | | Yazd | |  | 111 | 626566 | | Esfahan |
| 22 | 623123 | | Fars | |  | 112 | 626573 | | Esfahan |
| 23 | 623127 | | Fars | |  | 113 | 626699 | | Ilam |
| 24 | 623136 | | Azarbayjan-Gharbi | |  | 114 | 626706 | | Hamadan |
| 25 | 623161 | | Azarbayjan-Gharbi | |  | 115 | 626736 | | Khorasan |
| 26 | 623162 | | Azarbayjan-Gharbi | |  | 116 | 626747 | | Yazd |
| 27 | 623169 | | Gilan | |  | 117 | 626764 | | Yazd |
| 28 | 623176 | | Khorasan | |  | 118 | 626776 | | Khorasan |
| 29 | 623274 | | Bakhtaran | |  | 119 | 626814 | | Esfahan |
| 30 | 623291 | | Hamadan | |  | 120 | 626825 | | Esfahan |
| 31 | 623318 | | Yazd | |  | 121 | 626846 | | Yazd |
| 32 | 623338 | | Fars | |  | 122 | 626855 | | Markazi |
| 33 | 623344 | | Bakhtaran | |  | 123 | 626872 | | Fars |
| 34 | 623345 | | Kordestan | |  | 124 | 626881 | | Azarbayjan-Shargi |
| 35 | 623377 | | Kerman | |  | 125 | 626883 | | Fars |
| 36 | 623379 | | Kerman | |  | 126 | 626904 | | Azarbayjan-Shargi |
| 37 | 623382 | | Kerman | |  | 127 | 626908 | | Kerman |
| 38 | 623417 | | Sistan-Balouchestan | |  | 128 | 626924 | | Gilan |
| 39 | 623421 | | Azarbayjan-Shargi | |  | 129 | 626933 | | Hormozgan |
| 40 | 623428 | | Azarbayjan-Shargi | |  | 130 | 626943 | | Kerman |
| 41 | 623473 | | Ilam | |  | 131 | 626978 | | Esfahan |
| 42 | 623475 | | Ilam | |  | 132 | 627036 | | Khouzestan |
| 43 | 623503 | | Ilam | |  | 133 | 627038 | | Khouzestan |
| 44 | 623506 | | Bakhtaran | |  | 134 | 627043 | | Azarbayjan-Gharbi |
| 45 | 623507 | | Bakhtaran | |  | 135 | 627054 | | Gilan |
| 46 | 623508 | | Bakhtaran | |  | 136 | 627055 | | Zanjan |
| 47 | 623510 | | Azarbayjan-Gharbi | |  | 137 | 627057 | | Gilan |
| 48 | 623905 | | Bakhtaran | |  | 138 | 627066 | | Kerman |
| 49 | 623908 | | Bakhtaran | |  | 139 | 627072 | | Zanjan |
| 50 | 623909 | | Bakhtaran | |  | 140 | 627102 | | Zanjan |
| 51 | 623953 | | Bakhtaran | |  | 141 | 627103 | | Mazandaran |
| 52 | 623980 | | Hamadan | |  | 142 | 627189 | | Khorasan |
| 53 | 624215 | | Hamadan | |  | 143 | 627299 | | Yazd |
| 54 | 624240 | | Ilam | |  | 144 | 627356 | | Hormozgan |
| 55 | 624251 | | Ilam | |  | 145 | 627359 | | Markazi |
| 56 | 624315 | | Kordestan | |  | 146 | 627360 | | Kerman |
| 57 | 624378 | | Bakhtaran | |  | 147 | 627385 | | Bakhtaran |
| 58 | 624381 | | Bakhtaran | |  | 148 | 627399 | | Zanjan |
| 59 | 624576 | | Hamadan | |  | 149 | 627410 | | Azarbayjan-Shargi |
| 60 | 624580 | | Hamadan | |  | 150 | 627414 | | Bakhtaran |
| 61 | 624582 | | Hamadan | |  | 151 | 627416 | | Bakhtaran |
| 62 | 624585 | | Hamadan | |  | 152 | 627417 | | Bakhtaran |
| 63 | 624596 | | Hamadan | |  | 153 | 627423 | | Hamadan |
| 64 | 624804 | | Bakhtaran | |  | 154 | 627460 | | Khorasan |
| 65 | 624805 | | Bakhtaran | |  | 155 | 627484 | | Yazd |
| 66 | 624818 | | Ilam | |  | 156 | 627551 | | Azarbayjan-Shargi |
| 67 | 624837 | | Ilam | |  | 157 | 627587 | | Kordestan |
| 68 | 624838 | | Ilam | |  | 158 | 627616 | | Esfahan |
| 69 | 624846 | | Ilam | |  | 159 | 627688 | | Esfahan |
| 70 | 624849 | | Ilam | |  | 160 | 627723 | | Yazd |
| 71 | 624861 | | Ilam | |  | 161 | 627760 | | Azarbayjan-Shargi |
| 72 | 624863 | | Ilam | |  | 162 | 627787 | | Azarbayjan-Shargi |
| 73 | 624864 | | Ilam | |  | 163 | 627842 | | Kerman |
| 74 | 624894 | | Kordestan | |  | 164 | 627845 | | Sistan-Balouchestan |
| 75 | 624900 | | Kordestan | |  | 165 | 627849 | | Sistan-Balouchestan |
| 76 | 624901 | | Kordestan | |  | 166 | 627852 | | Sistan-Balouchestan |
| 77 | 624910 | | Hamadan | |  | 167 | 627853 | | Sistan-Balouchestan |
| 78 | 624911 | | Hamadan | |  | 168 | 627856 | | Mazandaran |
| 79 | 624925 | | Hamadan | |  | 169 | 627873 | | Zanjan |
| 80 | 624939 | | Tehran | |  | 170 | 627881 | | Azarbayjan-Shargi |
| 81 | 624941 | | Tehran | |  | 171 | 627883 | | Azarbayjan-Shargi |
| 82 | 624944 | | Tehran | |  | 172 | 627908 | | Markazi |
| 83 | 624946 | | Tehran | |  | 173 | 627948 | | Markazi |
| 84 | 624947 | | Tehran | |  | 174 | 627963 | | Hamadan |
| 85 | 624956 | | Tehran | |  | 175 | 627987 | | Zanjan |
| 86 | 624963 | | Tehran | |  | 176 | 627990 | | Bakhtaran |
| 87 | 624980 | | Gazvin | |  | 177 | 628084 | | Mazandaran |
| 88 | 624983 | | Gazvin | |  | 178 | 628088 | | Markazi |
| 89 | 624990 | | Gazvin | |  | 179 | 628114 | | Esfahan |
| 90 | 625047 | | Markazi | |  | 180 | 628189 | | Ilam |
| Genetic background: Cultivars | | | | | | | | | |
| No. | | Variety Name | | Introduced year | | | | Growth Habit | |
| 181 | | 4820 | | 1951 | | | | Spring | |
| 182 | | ADL | | 1976 | | | | Spring | |
| 183 | | AFLAK | | 2010 | | | | Spring | |
| 184 | | AKBARI | | 2006 | | | | Spring | |
| 185 | | AKOVA | | 1958 | | | | Winter | |
| 186 | | ALBORZ | | 1978 | | | | Spring | |
| 187 | | ALVAND | | 1995 | | | | Facultative | |
| 188 | | ARTA | | 2006 | | | | Spring | |
| 189 | | ARVAND1 | | 1974 | | | | Spring | |
| 190 | | ATRAK | | 1995 | | | | Spring | |
| 191 | | AZADI | | 1979 | | | | Facultative | |
| 192 | | AZAR | | 1957 | | | | Winter | |
| 193 | | AZAR2 | | 1997 | | | | Winter | |
| 194 | | BAHAR | | 2007 | | | | Spring | |
| 195 | | BAM | | 2006 | | | | Spring | |
| 196 | | BAYAT | | 1976 | | | | Spring | |
| 197 | | BEZOSTAYA | | 1969 | | | | Winter | |
| 198 | | BISTON | | 1980 | | | | Spring | |
| 199 | | CHAMRAN | | 1997 | | | | Spring | |
| 200 | | CHAMRAN2 | | 2013 | | | | Spring | |
| 201 | | DARAB1 | | 1980 | | | | Spring | |
| 202 | | DARAB2 | | 1995 | | | | Spring | |
| 203 | | DARYA | | 2006 | | | | Spring | |
| 204 | | DASTJERDI | | 1960 | | | | Spring | |
| 205 | | DAYHIM | | 1968 | | | | Spring | |
| 206 | | DEZ | | 2002 | | | | Spring | |
| 207 | | DN11 | |  | | | |  | |
| 208 | | FALAT | | 1990 | | | | Spring | |
| 209 | | FONG | |  | | | |  | |
| 210 | | FONTANA | |  | | | |  | |
| 211 | | GAHAR | | 1996 | | | | Spring | |
| 212 | | GASCOGNE | | 1994 | | | |  | |
| 213 | | GHODS | | 1988 | | | | Spring | |
| 214 | | GOLESTAN | | 1986 | | | | Spring | |
| 215 | | HAMOON | | 2002 | | | | Spring | |
| 216 | | HOMA | | 2009 | | | | Winter | |
| 217 | | INIA66 | | 1969 | | | | Spring | |
| 218 | | KARAJ1 | | 1974 | | | | Facultative | |
| 219 | | KARAJ2 | | 1974 | | | | Winter | |
| 220 | | KARAJ3 | | 1974 | | | | Winter | |
| 221 | | KARIM | | 2011 | | | | Spring | |
| 222 | | KAVEH | | 1980 | | | | Spring | |
| 223 | | KAVIR | | 1997 | | | | Spring | |
| 224 | | KHAZAR1 | | 1974 | | | | Spring | |
| 225 | | KOOHDASHT | | 2002 | | | | Spring | |
| 226 | | MAHDAVI | | 1995 | | | | Spring | |
| 227 | | MAROON | | 1991 | | | | Spring | |
| 228 | | MARVDASHT | | 1999 | | | | Spring | |
| 229 | | MIHAN | | 2010 | | | | Winter | |
| 230 | | MOGHAN1 | | 1974 | | | | Spring | |
| 231 | | MOGHAN2 | | 1974 | | | | Spring | |
| 232 | | MOGHAN3 | | 2006 | | | | Spring | |
| 233 | | MORVARID | | 2009 | | | | Spring | |
| 234 | | MV17 | | 1993 | | | | Winter | |
| 235 | | NAVID | | 1990 | | | | Facultative | |
| 236 | | NAZ | | 1978 | | | | Spring | |
| 237 | | NEISHABOUR | | 2006 | | | | Spring | |
| 238 | | NICKNEJAD | | 1995 | | | | Spring | |
| 239 | | OFOGH | | 2012 | | | | Spring | |
| 240 | | OHADI | | 2010 | | | | Winter | |
| 241 | | PANJAMO62 | | 1968 | | | | Spring | |
| 242 | | PARSI | | 2009 | | | | Spring | |
| 243 | | PISHGAM | | 2008 | | | | Facultative | |
| 244 | | PISHTAZ | | 2002 | | | | Spring | |
| 245 | | QABOOS | | 2014 | | | | Spring | |
| 246 | | RASHID | | 1968 | | | | Facultative | |
| 247 | | RIJAW | | 2011 | | | | Facultative | |
| 248 | | ROSHAN | | 1960 | | | | Spring | |
| 249 | | SEPAHAN | | 2006 | | | | Spring | |
| 250 | | SHAHI | | 1967 | | | | Winter | |
| 251 | | SHAHPASSAND | | 1942 | | | | Winter | |
| 252 | | SHAHRYAR | | 2002 | | | | Winter | |
| 253 | | SHANGHAI7 | |  | | | | Spring | |
| 254 | | SHIRAZ | | 2002 | | | | Spring | |
| 255 | | SHIROODI | | 1997 | | | | Spring | |
| 256 | | SIOSSON | | 1994 | | | |  | |
| 257 | | SIRVAN | | 2012 | | | | Spring | |
| 258 | | SISTAN | | 2006 | | | | Spring | |
| 259 | | SIVAND | | 2009 | | | | Spring | |
| 260 | | TAJAN | | 1995 | | | | Spring | |
| 261 | | TAKAB | | 2013 | | | | Winter | |
| 262 | | TOBARI66 | | 1969 | | | | Spring | |
| 263 | | TOUS | | 2002 | | | | Facultative | |
| 264 | | UROUM | | 2009 | | | | Winter | |
| 265 | | VEE/NAC | | 1997 | | | | Spring | |
| 266 | | ZAGROS | | 1996 | | | | Spring | |
| 267 | | ZARE | | 2010 | | | | Facultative | |
| 268 | | ZARRIN | | 1995 | | | | Facultative | |
| 181 | | 4820 | | 1951 | | | | Spring | |
| 182 | | ADL | | 1976 | | | | Spring | |
| 183 | | AFLAK | | 2010 | | | | Spring | |
| 184 | | AKBARI | | 2006 | | | | Spring | |
| 185 | | AKOVA | | 1958 | | | | Winter | |
| 186 | | ALBORZ | | 1978 | | | | Spring | |
| 187 | | ALVAND | | 1995 | | | | Facultative | |
| 188 | | ARTA | | 2006 | | | | Spring | |
| 189 | | ARVAND1 | | 1974 | | | | Spring | |
| 190 | | ATRAK | | 1995 | | | | Spring | |
| 191 | | AZADI | | 1979 | | | | Facultative | |
| 192 | | AZAR | | 1957 | | | | Winter | |
| 193 | | AZAR2 | | 1997 | | | | Winter | |
| 194 | | BAHAR | | 2007 | | | | Spring | |
| 195 | | BAM | | 2006 | | | | Spring | |
| 196 | | BAYAT | | 1976 | | | | Spring | |
| 197 | | BEZOSTAYA | | 1969 | | | | Winter | |
| 198 | | BISTON | | 1980 | | | | Spring | |
| 199 | | CHAMRAN | | 1997 | | | | Spring | |
| 200 | | CHAMRAN2 | | 2013 | | | | Spring | |
| 201 | | DARAB1 | | 1980 | | | | Spring | |
| 202 | | DARAB2 | | 1995 | | | | Spring | |
| 203 | | DARYA | | 2006 | | | | Spring | |
| 204 | | DASTJERDI | | 1960 | | | | Spring | |
| 205 | | DAYHIM | | 1968 | | | | Spring | |
| 206 | | DEZ | | 2002 | | | | Spring | |
| 207 | | DN11 | |  | | | |  | |
| 208 | | FALAT | | 1990 | | | | Spring | |
| 209 | | FONG | |  | | | |  | |
| 210 | | FONTANA | |  | | | |  | |
| 211 | | GAHAR | | 1996 | | | | Spring | |
| 212 | | Gascogne | | 1994 | | | |  | |
| 213 | | GHODS | | 1988 | | | | Spring | |
| 214 | | GOLESTAN | | 1986 | | | | Spring | |
| 215 | | HAMOON | | 2002 | | | | Spring | |
| 216 | | HOMA | | 2009 | | | | Winter | |
| 217 | | INIA66 | | 1969 | | | | Spring | |
| 218 | | KARAJ1 | | 1974 | | | | Facultative | |
| 219 | | KARAJ2 | | 1974 | | | | Winter | |
| 220 | | KARAJ3 | | 1974 | | | | Winter | |
| 221 | | KARIM | | 2011 | | | | Spring | |
| 222 | | KAVEH | | 1980 | | | | Spring | |
| 223 | | KAVIR | | 1997 | | | | Spring | |
| 224 | | KHAZAR1 | | 1974 | | | | Spring | |
| 225 | | KOOHDASHT | | 2002 | | | | Spring | |
| 226 | | MAHDAVI | | 1995 | | | | Spring | |
| 227 | | MAROON | | 1991 | | | | Spring | |
| 228 | | MARVDASHT | | 1999 | | | | Spring | |
| 229 | | MIHAN | | 2010 | | | | Winter | |
| 230 | | MOGHAN1 | | 1974 | | | | Spring | |
| 231 | | MOGHAN2 | | 1974 | | | | Spring | |
| 232 | | MOGHAN3 | | 2006 | | | | Spring | |
| 233 | | MORVARID | | 2009 | | | | Spring | |
| 234 | | MV17 | | 1993 | | | | Winter | |
| 235 | | NAVID | | 1990 | | | | Facultative | |
| 236 | | NAZ | | 1978 | | | | Spring | |
| 237 | | NEISHABOUR | | 2006 | | | | Spring | |
| 238 | | NICKNEJAD | | 1995 | | | | Spring | |
| 239 | | OFOGH | | 2012 | | | | Spring | |
| 240 | | OHADI | | 2010 | | | | Winter | |
| 241 | | PANJAMO62 | | 1968 | | | | Spring | |
| 242 | | PARSI | | 2009 | | | | Spring | |
| 243 | | PISHGAM | | 2008 | | | | Facultative | |
| 244 | | PISHTAZ | | 2002 | | | | Spring | |
| 245 | | QABOOS | | 2014 | | | | Spring | |
| 246 | | RASHID | | 1968 | | | | Facultative | |
| 247 | | RIJAW | | 2011 | | | | Facultative | |
| 248 | | ROSHAN | | 1960 | | | | Spring | |
| 249 | | SEPAHAN | | 2006 | | | | Spring | |
| 250 | | SHAHI | | 1967 | | | | Winter | |
| 251 | | SHAHPASSAND | | 1942 | | | | Winter | |
| 252 | | SHAHRYAR | | 2002 | | | | Winter | |
| 253 | | SHANGHAI7 | |  | | | | Spring | |
| 254 | | SHIRAZ | | 2002 | | | | Spring | |
| 255 | | SHIROODI | | 1997 | | | | Spring | |
| 256 | | SIOSSON | | 1994 | | | |  | |
| 257 | | SIRVAN | | 2012 | | | | Spring | |
| 258 | | SISTAN | | 2006 | | | | Spring | |
| 259 | | SIVAND | | 2009 | | | | Spring | |
| 260 | | TAJAN | | 1995 | | | | Spring | |
| 261 | | TAKAB | | 2013 | | | | Winter | |
| 262 | | TOBARI66 | | 1969 | | | | Spring | |
| 263 | | TOUS | | 2002 | | | | Facultative | |
| 264 | | UROUM | | 2009 | | | | Winter | |
| 265 | | VEE/NAC | | 1997 | | | | Spring | |
| 266 | | ZAGROS | | 1996 | | | | Spring | |
| 267 | | ZARE | | 2010 | | | | Facultative | |
| 268 | | ZARRIN | | 1995 | | | | Facultative | |

**Supplementary Table 2.** Climatic data in the studied environments

| Year | Month | Max Temperature ⁰C | Min Temperature ⁰C | Average Temperature ⁰C | Average rainfall, mm | Average relative humidity | Sunny hours | Evaporation, mm |
| --- | --- | --- | --- | --- | --- | --- | --- | --- |
| 2014 | November | 14.447 | 6.507 | 10.220 | 0.901 | 66.975 | 3.607 | 2.510 |
|  | December | 7.803 | -1.674 | 2.729 | 1.045 | 63.257 | 6.543 | 0.540 |
|  | January | 8.230 | -0.771 | 3.655 | 0.168 | 60.609 | 5.081 | 0.000 |
|  | February | 8.981 | -1.904 | 3.471 | 0.175 | 51.998 | 6.156 | 0.000 |
|  | March | 16.061 | 4.577 | 10.429 | 1.521 | 47.686 | 7.624 | 0.000 |
|  | April | 22.817 | 9.411 | 16.372 | 2.200 | 43.774 | 9.263 | 5.634 |
|  | May | 28.967 | 14.713 | 21.829 | 0.468 | 36.820 | 10.079 | 9.631 |
|  | June | 34.080 | 17.817 | 26.332 | 0.000 | 30.330 | 10.859 | 12.593 |
| 2015 | November | 12.590 | 3.427 | 7.637 | 1.047 | 61.060 | 6.013 | 1.627 |
|  | December | 10.541 | 1.580 | 5.900 | 0.735 | 60.875 | 6.497 | 0.000 |
|  | January | 10.280 | -0.061 | 5.093 | 0.094 | 50.951 | 6.168 | 0.000 |
|  | February | 11.731 | 0.940 | 5.970 | 0.883 | 58.149 | 5.592 | 0.000 |
|  | March | 15.184 | 3.393 | 9.258 | 0.972 | 52.696 | 6.832 | 0.000 |
|  | April | 22.989 | 9.610 | 16.466 | 0.473 | 35.339 | 9.423 | 6.150 |
|  | May | 29.548 | 13.671 | 21.837 | 0.301 | 35.061 | 8.387 | 8.783 |
|  | June | 35.868 | 19.373 | 27.650 | 0.007 | 26.449 | 10.937 | 12.803 |
| 2017 | November | 14.561 | 4.104 | 10.900 | 0.031 | 45.810 | 6.893 | 3.068 |
|  | December | 9.242 | -0.119 | 4.671 | 1.326 | 60.134 | 5.065 | 0.000 |
|  | January | 8.406 | -0.613 | 3.668 | 0.485 | 57.750 | 6.652 | 0.000 |
|  | February | 7.871 | -2.254 | 2.536 | 0.965 | 61.429 | 6.868 | 0.000 |
|  | March | 14.216 | 4.623 | 9.271 | 1.240 | 56.847 | 5.942 | 0.179 |
|  | April | 21.093 | 9.563 | 15.110 | 1.555 | 49.954 | 6.587 | 4.497 |
|  | May | 29.229 | 14.261 | 21.935 | 0.710 | 38.722 | 10.435 | 7.377 |
|  | June | 34.159 | 17.597 | 26.083 | 0.000 | 32.304 | 12.763 | 11.676 |
| 2018 | November | 17.080 | 6.383 | 11.520 | 0.021 | 43.479 | 6.960 | 3.189 |
|  | December | 12.303 | 1.652 | 6.671 | 0.152 | 50.419 | 7.226 | 0.000 |
|  | January | 9.077 | -0.055 | 4.052 | 0.640 | 54.476 | 6.526 | 0.000 |
|  | February | 10.739 | 2.039 | 6.464 | 1.094 | 64.755 | 5.829 | 0.000 |
|  | March | 20.558 | 8.377 | 14.652 | 0.455 | 38.952 | 7.303 | 0.000 |
|  | April | 19.983 | 7.793 | 13.633 | 1.527 | 51.413 | 7.563 | 6.714 |
|  | May | 25.513 | 12.061 | 18.432 | 1.841 | 54.907 | 8.287 | 6.161 |
|  | June | 33.807 | 17.347 | 25.583 | 0.241 | 37.492 | 11.100 | 11.143 |

**Supplementary Table 3.** Forty genotypes selected based on multi-trait stability index

| Genotype | WAASB |  | Genotype | WAASB |  | Genotype | WAASB |  | Genotype | WAASB |
| --- | --- | --- | --- | --- | --- | --- | --- | --- | --- | --- |
| 624381 | 0.4122 |  | 622311 | 0.8961 |  | 624990 | 1.0701 |  | 621704 | 1.1536 |
| CHAMRAN | 0.6371 |  | 627054 | 0.9246 |  | 627484 | 1.0852 |  | DAYHIM | 1.1552 |
| 624240 | 0.6532 |  | 627845 | 0.9537 |  | 624941 | 1.0865 |  | QABOOS | 1.1608 |
| 623162 | 0.7474 |  | NICKNEJAD | 1.0012 |  | TOUS | 1.0891 |  | 626260 | 1.1956 |
| 623503 | 0.763 |  | DASTJERDI | 1.0086 |  | FONTANA | 1.0904 |  | 627963 | 1.2139 |
| 624596 | 0.7845 |  | 626814 | 1.023 |  | 623908 | 1.1048 |  | 627948 | 1.2175 |
| 626904 | 0.8742 |  | 623475 | 1.0244 |  | 627043 | 1.1086 |  | 623382 | 1.2194 |
| SEPAHAN | 0.8807 |  | SISTAN | 1.032 |  | 627760 | 1.1139 |  | 627359 | 1.2218 |
| 627066 | 0.8889 |  | 623953 | 1.0429 |  | 624849 | 1.122 |  | VEE/NAC | 1.2328 |
| 625810 | 0.8907 |  | 625123 | 1.0431 |  | 623344 | 1.1458 |  | AZAR2 | 1.2552 |


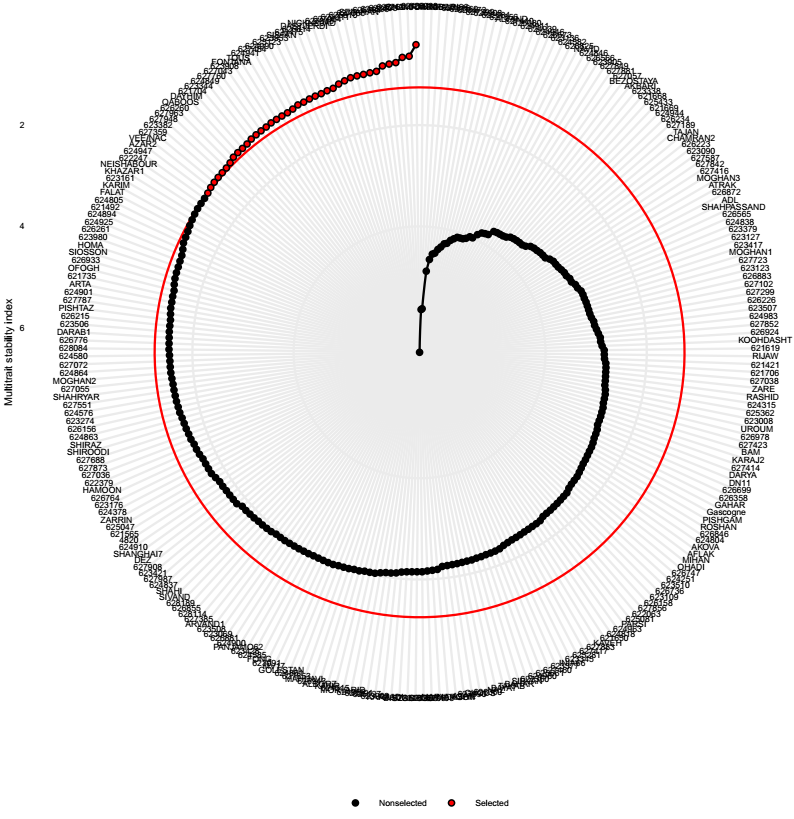


**Supplementary Fig 1.** Genotypes selected by multi-trait stability index applied on grain yield, number of grains, and spike weight


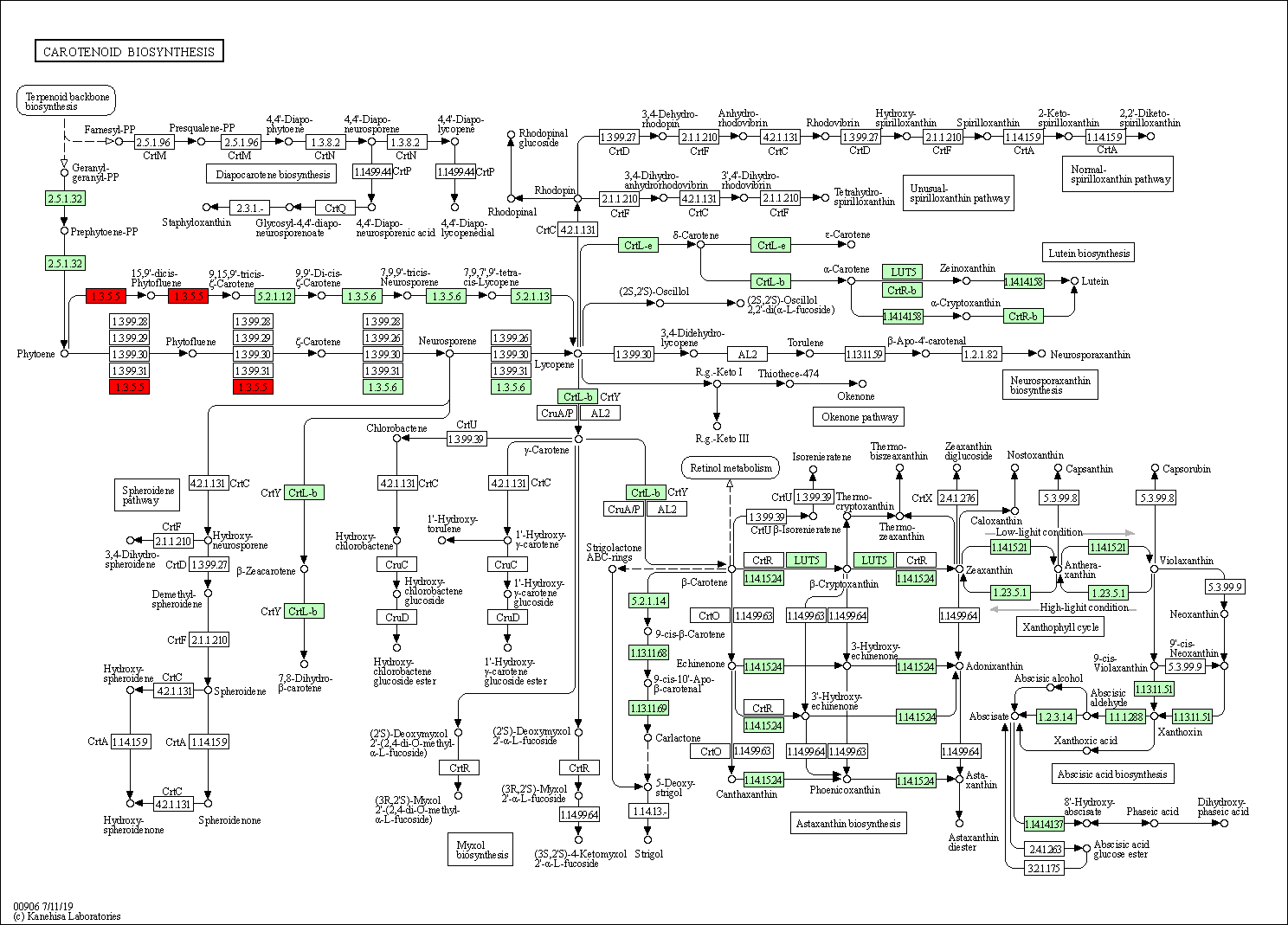


**Supplementary Fig 2.** The KEGG pathway of carotenoid biosynthesis


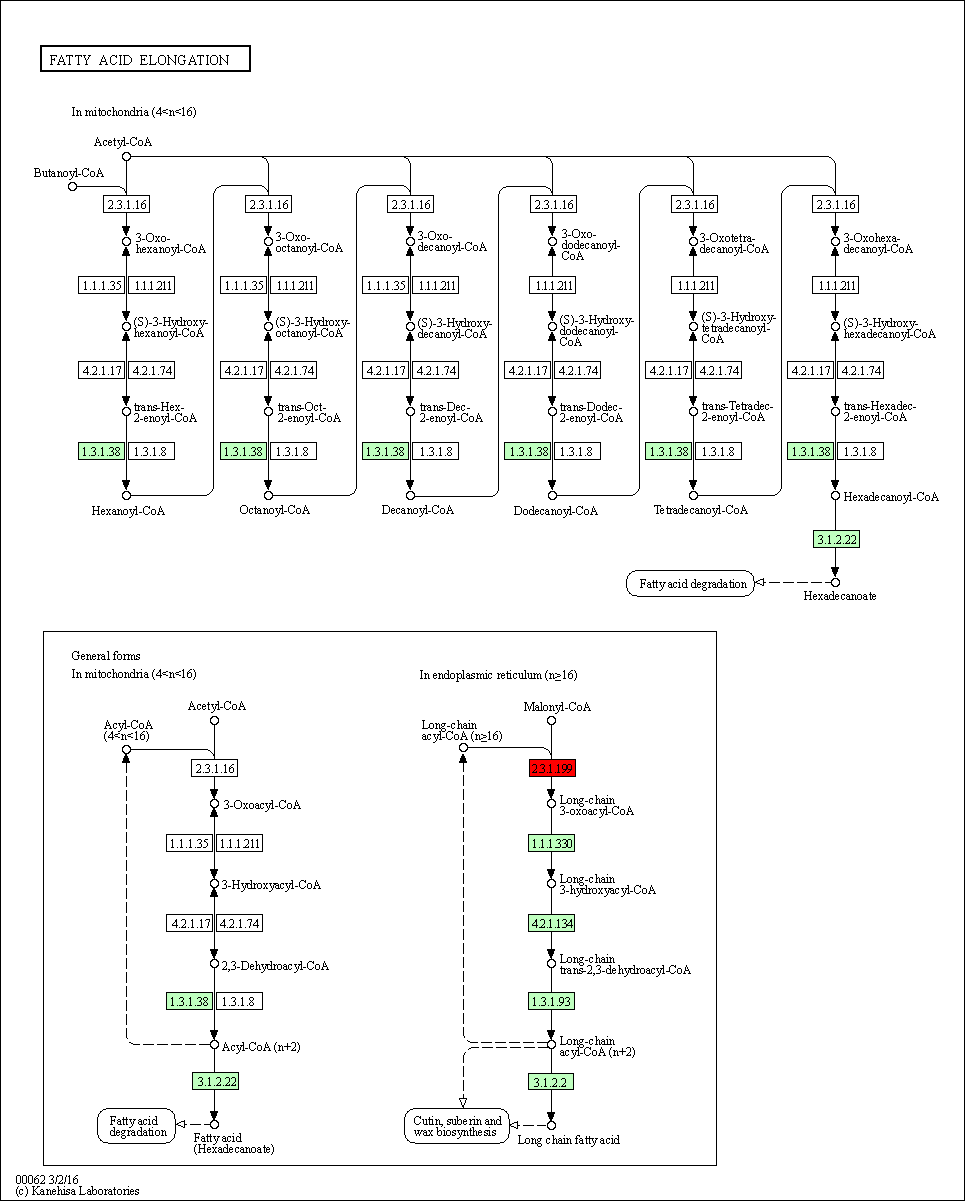


**Supplementary Fig 3.** The KEGG pathway of fatty acid elongation


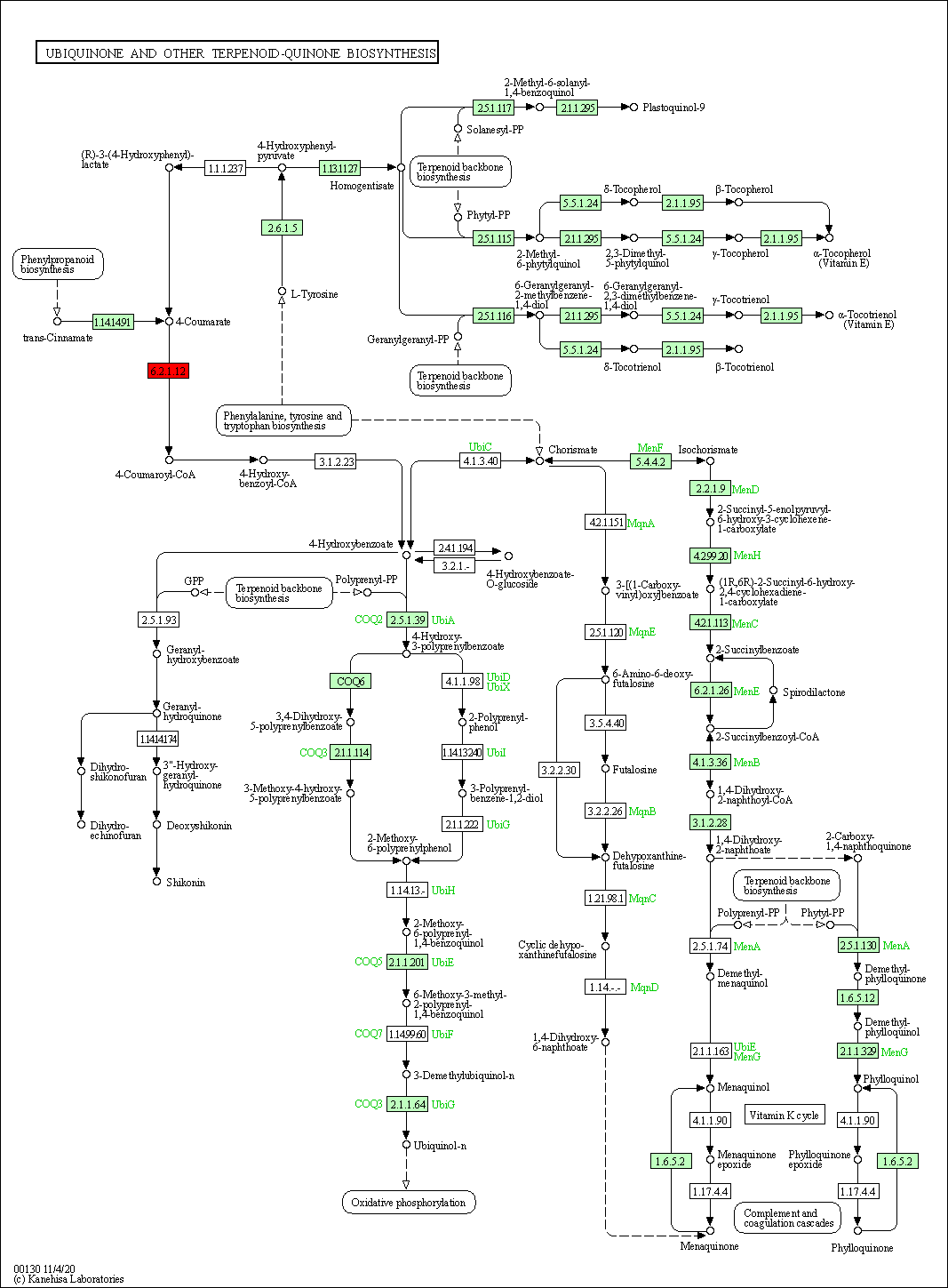


**Supplementary Fig 4.** The KEGG pathway of Ubiquinone and other terpenoid-quinone biosynthesis
